# Microbial Degradation of Lobster Shells to Extract Chitin Derivatives for Plant Disease Management

**DOI:** 10.3389/fmicb.2017.00781

**Published:** 2017-05-05

**Authors:** Gayathri Ilangumaran, Glenn Stratton, Sridhar Ravichandran, Pushp S. Shukla, Philippe Potin, Samuel Asiedu, Balakrishnan Prithiviraj

**Affiliations:** ^1^Marine Bio-products Research Laboratory, Department of Plant, Food and Environmental Sciences, Faculty of Agriculture, Dalhousie University, TruroNS, Canada; ^2^Department of Plant, Food and Environmental Sciences, Faculty of Agriculture, Dalhousie University, TruroNS, Canada; ^3^Station Biologique de RoscoffRoscoff, France

**Keywords:** biodegradation, lobster shells, *Streptomyces*, chitinase, plant defense

## Abstract

Biodegradation of lobster shells by chitinolytic microorganisms are an environment safe approach to utilize lobster processing wastes for chitin derivation. In this study, we report degradation activities of two microbes, “S223” and “S224” isolated from soil samples that had the highest rate of deproteinization, demineralization and chitinolysis among ten microorganisms screened. Isolates S223 and S224 had 27.3 and 103.8 protease units mg^-1^ protein and 12.3 and 11.2 μg ml^-1^ of calcium in their samples, respectively, after 1 week of incubation with raw lobster shells. Further, S223 contained 23.8 μg ml^-1^ of *N*-Acetylglucosamine on day 3, while S224 had 27.3 μg ml^-1^ on day 7 of incubation with chitin. Morphological observations and 16S rDNA sequencing suggested both the isolates were *Streptomyces*. The culture conditions were optimized for efficient degradation of lobster shells and chitinase (∼30 kDa) was purified from crude extract by affinity chromatography. The digested lobster shell extracts induced disease resistance in *Arabidopsis* by induction of defense related genes (*PR1* > 500-fold, *PDF1.2* > 40-fold) upon *Pseudomonas syringae* and *Botrytis cinerea* infection. The study suggests that soil microbes aid in sustainable bioconversion of lobster shells and extraction of chitin derivatives that could be applied in plant protection.

## Introduction

American lobster (*Homarus* *americanus*) is Canada’s most valuable fishery resource and exported around the world. Annual lobster landings in the waters of Atlantic Canada show an upward trend over recent decades and remain at one of the highest levels recorded in 100 years and 82,741 tons of lobster valued at $2 billion CAD were exported from this region in 2015 (Fisheries and Oceans Canada, 2016). Lobsters are processed into product forms (cooked or raw meat) except for a small faction being sold live. The processing begins with removal of the hard shell (exoskeleton) and huge volumes of it are generated during commercial scale processing. The bulk of shells are usually dumped in sea or landfills along the shoreline, which exceeds the rate of natural recycling process and threatens the ecosystem balance of coastal areas due to foul smell and release of biogenic amines from decay (Kelleher, 2005; Xu et al., 2008). The present waste management practices pose a serious environmental challenge to lobster and other crustacean processing plants. The exoskeleton of American lobster is composed of chitin, proteins, calcium, carotenoids and traces of other minerals and organic compounds. Chitin constitutes about 75% organic fraction of the exoskeleton (Fabritius et al., 2009). Chitin and its deacetylated derivative chitosan have diverse chemical and biological applications (Muzzarelli, 2009). Industrial production of chitin is carried out through acid, alkali and heat treatments of crustacean shells to remove protein and calcium constituents, which deposit hazardous effluent residues (Percot et al., 2003). Hence, utilization of the vast amount of waste shells discarded from lobster processing facilities and formulating an eco-friendly substitute to the physicochemical extraction of chitin from the shells are environmental obligations in the plight of curtailing pollution and maintaining sustainability.

Chitinolytic microorganisms play an important biogeochemical role by recycling chitin, the second most abundant natural polysaccharide made of β-1,4 *N*-Acetylglucosamine units. Those microbial populations may assimilate chitin as the sole source of carbon and nitrogen. Microorganisms found in chitin rich niches enzymatically digest the shells by secreting chitinases, proteases, other enzymes and organic acids. Endo and exochitinases hydrolyze chitin to *N*-Acetylglucosamine and chitin deacetylase cleaves chitin to chitosan and acetyl group (Gooday, 1990). Chitinases are also synthesized by diverse plant tissues and plant chitinases are primarily involved in defense against biotic stress (Mauch et al., 1988; Punja and Zhang, 1993). Chitinolytic bacteria and fungi are used as biocontrol agents in agriculture since they digest chitin and its derivatives, which are the major constituents of bacterial peptidoglycan, fungal cell wall and insect cuticle (Ordentlich et al., 1988). Chitin extraction through microbial degradation of crustacean shells has been explored in earlier studies. Lactic acid fermentation improved ensilation of shrimp heads with added whey, lignocellulose and starch (Fagbenro, 1996). Microorganisms present in crustacean gut microbiota and probiotic curd were used to separate chitin from shrimp waste (Rao et al., 2000). Successive microbial demineralization and deproteinization of crab and shrimp shells yielded liquid fraction rich in proteins and minerals and an insoluble chitin fraction, which was retained in the sediment (Jung et al., 2007; Xu et al., 2008). Since previous researches centered on shells of other crustaceans such as shrimp and crab but not lobster yet, this study attempted to harness the potential of waste lobster shells through biodegradation in the given context of local environment and economy.

Chitin derivatives obtained from crustacean shells are used in agriculture to promote plant growth and control plant diseases. Their diverse mechanism of actions result in direct antimicrobial activity, synergistic effect on beneficial microbes, elicitation of plant defense responses and stimulation of plant metabolism (El Hadrami et al., 2010; Ramírez et al., 2010). Chitosan has been reported to inhibit growth of a wide range of bacteria and fungi (Rabea et al., 2003; El Hadrami et al., 2010; Xia et al., 2011; Sharp, 2013). The antimicrobial activity of chitosan is due to its cationic properties, which interrupt potassium signaling in pathogens. Moreover, chitosan disrupts membrane integrity of vacuoles and endomembrane organelles in fungal pathogens (Rabea et al., 2003; Sharp, 2013). O’Herlihy et al. (2003) showed inhibitory activity of chitosan against oomycete pathogens such as *Phytophthora capsici* and *P. infestans*. The polymeric form of chitin exerts antimicrobial activity by creating barrier films, chelating mineral nutrients and preventing the release of mycotoxin from pathogens (Sudarshan et al., 1992; Sharp, 2013). The chitin derivative obtained from ground shrimp waste controlled *Streptomyces scabies*, a causal agent for scab disease on potato tubers (Vruggink, 1970). Chitin oligosaccharides have been reported to act as pathogen associated molecular patterns (PAMPs) due to their structural similarity to the constituents of pathogen cell wall in many plant pathosystems. PAMPs are recognized by host transmembrane pattern recognition receptors (PRRs), which signal defense pathways of induced systemic resistance (ISR) and systemic acquired resistance (SAR) (Eckardt, 2008; Zipfel, 2009). The chitin derivatives thus obtained from the microbial degradation of lobster shells can be used as elicitors of innate and systemic immune responses in plants (Benhamou, 1996; Jones and Dangl, 2006). Therefore, when actual pathogen incidence occurs the plant disease resistance mechanisms confer enhanced protection against it. The objectives of this study were met through investigation of microbial degradation of lobster shells and potential applications of the metabolized extracts in plant protection. Lobster shells were inoculated with microbes isolated from soil and culture conditions were optimized for their efficient degradation. The chitinous extracts collected from microbial digestion were applied to induce disease resistance in *Arabidopsis thaliana* against *Pseudomonas syringae* pv. *tomato* DC3000 and *Botrytis cinerea*.

## Materials and Methods

### Isolation and Screening of Microorganisms for Lobster Shell Degradation

Lobster shells (cooked and raw) were procured from Aquashell Holdings Inc., Wallace, NS, Canada. The shells were washed, dried, ground and sieved (1 mm sieve) and used as lobster shell powder (LSP). Soil samples were collected at Jost vineyards, Malagash, NS, Canada, where the soil has been amended with lobster shells as a nutrient source. Microbe isolation from the samples was performed by serial dilution method on agar plates containing 0.5% (w/v) LSP as sole carbon source in M9 minimal buffer (excluding CaCl_2_) at pH 7 with 2% (w/v) agar and incubated at 25°C. Morphologically distinct colonies were sub-cultured and maintained on LSP agar plates. Strains of *Bacillus subtilis*, *Pseudomonas fluorescens*, *Trichoderma harzianum*, and *Lactobacillus acidophilus* were previously studied for crustacean shell waste degradation (Sini et al., 2007; Phuvasate and Su, 2010; Das et al., 2012; Lee and Kim, 2015). These microbes (revived from laboratory stock cultures; *B. subtilis* NRS 231, *P. fluorescens* 271, *T. harzianum* AB 63-3 and *L. acidophilus* Scav) were screened for lobster shell degradation activity along with the soil isolates. All the microorganisms were grown in M9 buffer with 0.5% (w/v) LSP at 25°C for 48 h by inoculating a loop of scrapped colonies or mycelium grown on agar plates and this propagation method was followed to obtain the starter inoculum for all the other experiments. 1 mL of starter culture was incubated with 1% (w/v) LSP in M9 buffer at 25°C, 150 rpm and samples were collected by centrifugation (6,000 × *g*, 20 min) for assaying degradation activity. Microbial deproteinization and demineralization of lobster shells were determined by protease activity using phenol quantification method of tyrosine (Folin and Ciocalteu, 1927) and calcium ions measured in Atomic absorption spectrometer (Varian AAS), respectively. Chitin (from shrimp shells, Sigma) was added to M9 buffer instead of LSP and chitinolysis was determined by quantifying *N*-Acetyl glucosamine (GlcNAc) content of the samples with 3,5-Dinitrosalicylic acid reagent (Monreal and Reese, 1969). Based on deproteinization, demineralization and chitinolysis activity, two soil isolates (S223 and S224) were selected for further experiments on lobster shell degradation.

### Identification of Soil Microbes

Morphological observations and nutrients utilization tests of the two soil microbes, S223 and S224 were performed according to the procedures described in the International Streptomyces Project (Shirling and Gottlieb, 1966). Genomic DNA of the actinomycetes were extracted following standard DNA isolation protocol (Sambrook et al., 2012). The universal eubacterial primers: 8F (5′-AGAGTTTGATCCTGGCTCAG-3′) and 1492R (5′-GGTTACCTTGTTACGACTT-3′) were used for the amplification of 16S ribosomal DNA gene (Salaun et al., 2010). Purified DNA preparations were run in ABI 3130xl capillary sequencer (Applied Biosystems) at the Station Biologique de Roscoff, Genopole Ouest, Roscoff, France. The 16S rDNA sequences were edited using DNAMAN v. 4.15 software, and compared with sequences deposited in public databases using BLAST (Basic Local Alignment Search Tool).

### Optimization of Microbial Culture Conditions

Several factors affecting the degradation process of lobster shell were optimized for the growth of the two *Streptomyces sp.* Based on preliminary trials, the standard conditions were setup as: 1% (w/v) LSP in M9 buffer (pH 7) inoculated with 1% (v/v) microbe culture incubated at 25°C, 150 rpm for 14 days and modified accordingly for each factor tested. Treatments were run in triplicates and the experiment analyzed one factor at a time. Deproteinization and demineralization activities were measured as described above. GlcNAc was quantified as described by Reissig et al. (1955) to determine chitinolysis of lobster shells using p-Dimethylaminobenzaldehyde reagent.

### Preparation of Crude Extract from Culture Filtrates

*Streptomyces sp*. S223 and S224 were grown in 100 mL M9 buffer (pH 7) containing 1 g of raw LSP at 25°C, 150 rpm for 14 days in 250 mL Erlenmeyer flasks closed with cotton plugs. Supernatants were collected after centrifugation (6,000 × *g*, 20 min) and filter sterilized (0.2 μm) for use as crude extracts.

### Gel Diffusion Assay

Crude extracts were added to glycol chitin embedded agarose gel plates (pH 7) prepared as described by Velasquez and Hammerschmidt (2004). Supernatants from 7 day old cultures of S223 and S224 grown in yeast extract and malt extract (YEME) broth were included as controls. Dilutions of *S. griseus* chitinase (Sigma^®^) served as the standard. After the assay, plates were photographed under long wave UV transillumination (BioRad GelDoc). A contrast developed between fluorescent background and dark circular zones indicated chitin hydrolysis by chitinase.

### Chitinase Purification

The protein in crude extract was collected by centrifugation (14,000 × *g*, 30 min) following precipitation with ammonium sulfate (85% saturation). The pellet was suspended in 50 mM sodium phosphate buffer (pH 7.0) and dialyzed against the same buffer overnight. Chitinase was purified by affinity chromatography with colloidal chitin as substrate (Escott et al., 1998). The samples were concentrated using Amicon PM30 (MWCO 30kDa) ultrafiltration centrifugal tubes (Brzezinska et al., 2013). At each purification step, chitinase activity was measured using a nitrophenol based chitinase assay kit (Sigma). One unit of enzyme activity is defined as the amount of enzyme releasing 1 μM NP min^-1^ at pH 4.8 at 37°C. The protein content of samples was measured by Bradford (1976) method. Molecular weight of the purified chitinase was determined by SDS-PAGE using 12% gel in Tris-glycine buffer, pH 8.3 (Laemmli, 1970). Chitinase of *S. griseus* was used as standard reference. The gel was stained in Coomassie brilliant blue R-250 and following destaining, protein bands were photographed under bright field illumination.

### Antimicrobial Activity against *Pseudomonas syringae* and *Botrytis cinerea*

The antibacterial activity of extract was tested against *P. syringae* pv. *tomato* (*Pst*) DC3000 by microdilution method as described by Karaman et al. (2003). *Pst* DC3000 was grown in Kings B broth overnight. Culture of *Pst* DC3000 (0.01 O.D. at λ_600 nm_) was added in 1:1 ratio with crude extracts in a 96-well plate and incubated at 28°C. The antifungal potential of crude extracts against *B. cinerea* (laboratory stock) was evaluated by similar method. *B. cinerea* was grown on one-half strength potato dextrose agar. Fungal spores were collected by adding potato dextrose broth to the agar surface and filtered through sterile cheesecloth. The spore suspension (10^4^/mL) was added in 1:1 ratio with crude extracts in a 96-well plate and incubated at 25°C. Optical density for *Pst* DC3000 (λ_600 nm_) and *Botrytis* (λ_595 nm_) were read after 24 h and 48 h (Cytation 3, Biotek). Inhibition ratio was calculated according to the following equation:

$$\text{Inhibition ratio = }\frac{\text{absorbance of control }(\text{water})\;-\text{ absorbance of tested extracts}}{\text{absorbance of control }(\text{water})}$$

### Pathogenicity Studies of Arabidopsis in Growth Chamber Conditions

Arabidopsis wild type Col-0 seeds (Lehle seeds, USA) were planted on Jiffy peat pellets and transferred to growth chambers set at 22°C under light intensity of 150–200 E m^-2^ sec^-1^ in 16:8 h day-night cycle. Three to four weeks old plants were treated by foliar spray (flow-rate 6 mL/min) with crude lobster shell extracts (test), water (control) or chitosan (positive control) and inoculated with pathogens after 48 h of treatment application. Chitosan, the deacetylated derivative of chitin is known as an antimicrobial agent and an elicitor of defense responses in plants. Chitosan has been applied as a protective agent against soil-borne diseases in seeds, foliar spray in plants and used as a soil amendment (Lafontaine and Benhamou, 1996). The experiments were repeated twice with equal number of replicates.

### Phenotypic Observation of Disease Severity

After 48 h of treatment, the fully expanded rosette of Arabidopsis plants were dip inoculated with *Pst* DC3000 (0.05 O.D. at λ_600 nm_) as described by Bisgrove et al. (1994). Plants were observed for water-soaked spreading lesions with chlorosis symptoms (Preston, 2000) on 5th day post-inoculation. Nine randomly selected Arabidopsis plants were pooled into three replications and leaf samples were collected at 24, 48, 72, and 96 h after inoculation, weighed and surface sterilized with 75% (v/v) ethanol. The leaves were macerated using micropestles in sterile water and suspensions were plated on Kings B medium containing rifampin (25 μg/mL). The plates were incubated at 28°C for 48 h and number of colony forming units (cfu/mg fresh weight) was counted (Subramanian et al., 2011). Six to eight leaves per plant were spot inoculated with 20 μL *Botrytis* spore suspension (10^6^/mL). *Botrytis* causes water-soaked lesion that turns necrotic (Dean et al., 2012) and the size of lesions formed was measured on 3rd and 5th day post-inoculation. All the plants were kept under 100% relative humidity to encourage disease incidence. The disease severity experiment was repeated twice with three plants.

### Quantitation of Enzyme Activity and Gene Expression

For biochemical and gene expression studies, *Arabidopsis* plants were grown in similar conditions and subjected to infection as described above except that the pathogen inoculum were foliar sprayed rather dip or spot inoculation. A control group of plants were treated with lobster shell extracts to observe induced defense responses without any pathogen inoculation and they were noted as mock (no infection). Nine plants were randomly selected and leaves from three plants were pooled and constituted a replicate. The experiment was repeated twice. Leaf tissues were excised at 24 and 48 h post-mock treatment and inoculation (*Pst* DC3000 or *Botrytis*), weighed (∼200 mg) and snap frozen in liquid nitrogen immediately and stored at -80°C. For the estimation of enzyme activity, frozen leaf tissues were ground and collected in extraction buffer (1 mL) containing 0.3 g/L polyvinylpyrrolidone. The homogenate was centrifuged 16,000 × *g* (Beckman Coulter^TM^ Microfuge^®^) for 30 min at 4°C and the supernatant was used as the crude enzyme extract. Protein content of the extracts was measured using Bradford (1976) method with BSA as standard. Chitinase was quantified according to the procedure of Dietrich et al. (2004) and chitinase from *S. griseus* was used as the standard. Ground leaf tissues were collected in 25 mM sodium acetate buffer (pH 5.0). The reaction preparation contained crude enzyme (100 μL) and glycol chitin (0.1%) in 100 mM Na-acetate buffer (500 μL). The reaction was incubated for 30 min at 37°C and potassium ferricyanide (1 mL) was added to the reaction tubes, which results in development of yellow color and the samples were measured at λ_420 nm_ (Cytation 3, Biotek). Phenylalanine ammonia lyase activity was measured as μmol cinnamic acid min^-1^ mg protein^-1^ (Subramanian et al., 2011) using known cinnamic acid standards. Leaf tissues were homogenized in 25 mM ice-cold borate buffer (pH 8.8). The reaction mixture containing extract (200 μL) and 15 mM L-phenylalanine in 25 mM borate buffer (800 μL) was incubated for 1 h at 37°C. The cinnamic acid produced during the reaction was read at λ_290 nm_ (Cary^®^ UV 100 visible spectrophotometer).

Total RNA was extracted from the frozen leaf tissues by monophasic extraction (Chomczynski and Mackey, 1995) using Trizol^®^ reagent (Life technologies) and chloroform. RNA content in the samples were quantified in a NanoDrop spectrophotometer (ThermoScientific^®^) and analyzed by electrophoresis on 1% (w/v) agarose gel, visualized under UV *trans* illiumination (BioRad GelDoc^TM^). RNA (2 μg samples) was treated with RNase free DNase I (Promega^®^) for 30 min in a thermal cycler at 60°C and stop solution was added. Reverse transcription of RNA to cDNA was performed using high capacity cDNA Reverse transcription kit (Applied Biosystems). cDNA synthesis was confirmed by PCR amplification with *Actin* primers and electrophoresis of the samples on agarose gel. The 10 μL reaction mix loaded on to qPCR reaction plates (Applied Biosystems) contained 2 ng of cDNA synthesized with 300 nM gene specific primers (Invitrogen^TM^, Supplementary Table S1) and 5 μL GoTaq SYBR^®^ Green master mix (Promega^®^). The reactions were run on a StepOne Real-Time PCR system according to the manufacturer protocols (Applied Biosystems). Relative expression levels of four defense genes of interest, *PR1*, *PR3*, *PDF1.2*, and *ICS1* were calculated using ΔΔCt method with *Actin* as endogenous control.

### Statistical Analysis

Experiments were setup in a completely randomized design and results are expressed as mean ± standard error (SE). ANOVA (Analysis of variance) of the data was performed using SAS v. 9.3 statistical software (SAS Institute Inc., Cary, NC, USA), with general linear model or repeated measures model at a 95% confidence interval and 5% level of significance. When *P*-value was less than 0.05, multiple means comparison by Tukey’s HSD (honest significant difference) method was used to find means that are significantly different from others.

## Results and Discussion

Soil and sediments are widely used for isolation of chitinolytic bacteria (Chen and Li, 1993). These microbes are capable of decomposing chitin under aerobic and anaerobic conditions (Brzezinska et al., 2014). In this study, chitinolytic microorganisms were isolated from rhizospheric soils of a vineyard where lobster shells were applied as soil amendment (Ilangumaran, 2014).

### Microorganisms Isolated and Screened for Lobster Shell Degradation

Six microorganisms were subsequently isolated from soil samples on LSP agar plates based on colony growth, distinct morphology and formation of clearing zone (Supplementary Figure S1). They were named S113, S223, S224, S2231 (actinomycetes), S23, S232 (bacteria). Bacterial colonies were slimy and actinomycetes had raised white filamentous colony. The isolated microbes and *B. subtilis*, *P. fluorescens*, *L. acidophilus*, *T. harzianum* were tested for their ability to grow on chitin, raw and cooked lobster shells and colony diameter was measured on 7th day of incubation. *T. harzianum* covered the entire plate within 3 days of incubation and so, its diameter couldn’t be taken into account. The actinomycetes showed profuse growth on raw LSP.

**FIGURE 1:**
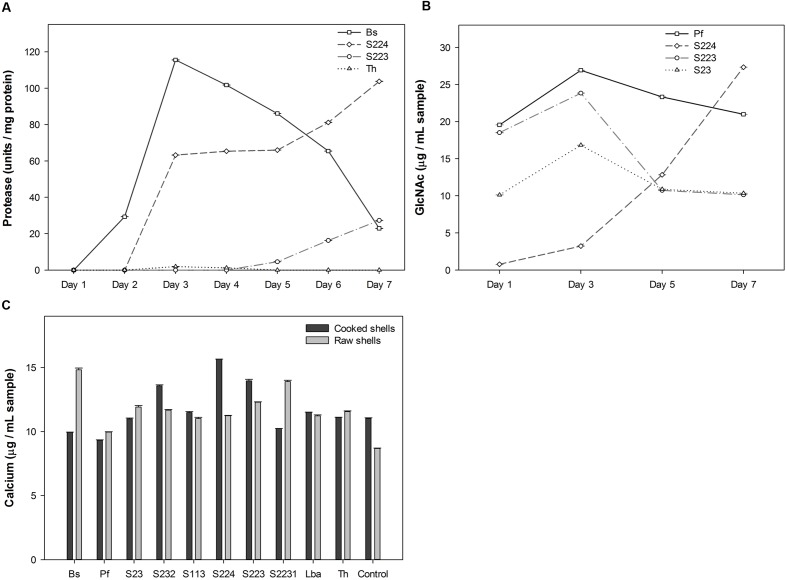
**Screening for microbial degradation of lobster shells.** **(A)** Protease activity measured in the culture supernatants of microbes grown in raw lobster shells from one to 7 days after incubation, using Folin-Ciocalteu’s phenol reagent. Four microorganisms exhibited deproteinization activity among ten screened. **(B)** Calcium content measured in the culture supernatants of microbes grown in cooked and raw lobster shells on 7 days after incubation by Atomic absorption spectroscopy. **(C)** GlcNAc (*N*-Acetyl glucosamine) quantified in the culture supernatants of microbes where pure chitin was used as the carbon source. Result of four microorganisms with significantly high GlcNAc content is shown. Values represent mean ± SE (*n* = 3; *P* < 0.05). Bs – *B. subtilis*, Pf – *P. fluorescens*, Lba – *L. acidophilus*, Th – *T. harzianum*, Control – no inoculation.

Deproteinization, demineralization and chitinolysis activities were used to select candidate microbes (**Figure 1** and Supplementary Data Sheet 1). *B. subtilis*, *T. harzianum*, S223, and S224 exhibited deproteinization activity of raw lobster shells. *B. subtilis* known for its ability to deproteinize crustacean shells (Sini et al., 2007), secreted 115.53 protease U mg^-1^ protein on 3rd day of incubation with raw lobster shells. Two actinomycetes, S223 and S224 showed a gradual increase in protease activity over the incubation period (**Figure 1A**). Only *B. subtilis* showed protease activity in cooked lobster shells and the absence of detectable activity by other microbes is due to the relatively low protein content of the shells (Waterman, 1991). The microbes tested exhibited significant (*P* = 0.0002) differences in their demineralization activity. Some microbes demineralized cooked shells predominantly while others demineralized uncooked shells (**Figure 1B**). The calcium content was higher in S224 (15.7 μg mL^-1^) and *B. subtilis* (14.8 μg mL^-1^) on incubation with cooked and raw LSP, respectively. Cooked shells contain more calcium per weight than that of raw shells (Supplementary Figure S2) and so, the release of Ca^2+^ into the medium was expected to be higher in digestion of cooked shells but the difference was not significant (*P* = 0.87) in the experiment. Deproteinization of 90–94% of shrimp shells was achieved by digesting the shells with *Serratia marcescens* (Jung et al., 2007). *B. licheniformis*, capable of deproteinising shrimp shells exhibited a protease activity of 60units/ml (Waldeck et al., 2006). Organic acids secreted by the microbes erode calcium from the shells and dissociates Ca^2+^ ions in to the solution, which in turn would form insoluble calcium salts of oxalate, formate or lactate (Oh et al., 2007). Ensilage of shrimp shells with lactic acid bacteria had resulted in > 99% demineralisation of the shells in 2–3 days of incubation, by precipitation of calcium to calcium lactate, which lowered the pH and induced activation of proteases (Xu et al., 2008). Hence, quantification of calcium reflects the quantity of calcium ions present in the sample solution at any given time point. Analysis of the digested sediments would reveal the amount of protein digested by enzymes and calcium precipitated by organic acids.

Pure chitin was used as the carbon source to screen chitinolytic microorganisms since the pigments presents in the lobster shells interfered with the colorimetric detection of GlcNAc in the samples using 3,5-Dinitrosalicylic acid reagent (data not shown). *P. fluorescens*, S23, S224 and S223 had significantly (*P* < 0.0001) higher concentration of GlcNAc in their samples than other microbes (**Figure 1C**). The GlcNAc content was variable throughout the sampling period due to compound fragmentation to simpler molecules such as glucose, amine and acetyl groups. S224 had the highest quantity (27.3 μg mL^-1^) on 7th day of incubation while S223 (23.8 μg mL^-1^) and *P. fluorescens* (26.9 μg mL^-1^) attained their maximum concentration on 3rd day of incubation. Based on the degradation activity screening, S223 and S224 isolated from soil samples exhibiting comparatively greater deproteinization, demineralization and chitinolysis were selected and studied in further experiments on the biological breakdown of lobster shells. Research studies have been published on *B. subtilis, P. fluorescens, T. harzianum*, and *L. acidophilus* degrading other crustacean shells (shrimp and crab). The soil isolates (S223 and S224) are unknown of their chitinolytic metabolism and expected to open new perspectives on lobster shell degradation.

### Soil Isolates Belong to *Streptomyces* Genus

Preliminary identification revealed that isolates S223 and S224 are Gram-positive, filamentous, spore bearing actinomycetes (Supplementary Figure S3). Dense network of filaments bear conidia like spores on aerial hypha, which are flexible, slender and branched. Spores are round shaped, endogenous in origin and form smooth chains. Other characters observed are powdery mass on the surface, raised colonies with crates, distinct earthy odor and clearing zones on starch agar. The isolates produced melanin pigments in peptone iron and tyrosine agar (Supplementary Figure S4). Morphological characteristics implied that the soil isolates belong to *Streptomyces* genus (Waksman and Henrici, 1943; Shirling and Gottlieb, 1966). Based on the alignment of 16S rDNA sequences, the isolates S223 and S224 shared 99% identity to *S. coelicolor* DSM 40233 and 96% identity *Streptomyces sp.* PGPA39, respectively, and the sequences were submitted to NCBI GenBank database (Accession numbers: KY818664 and KY818663). Adaptability of the microorganisms to grow at different temperatures and pH were observed on lobster shell powder agar plates and the colony growth indicated conducive temperature at 25–30°C and pH 7 (Supplementary Figure S5).

### Culture Conditions Optimized for Efficient Degradation of Lobster Shells

**FIGURE 2:**
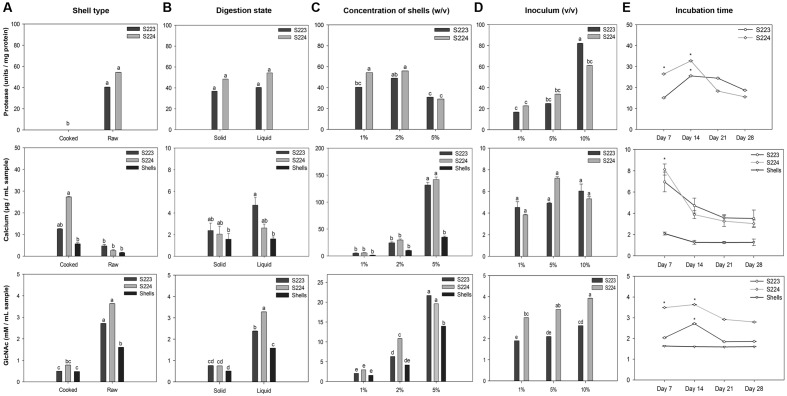
**Optimization of microbial culture conditions for lobster shell degradation.** Factors **(A)** Type of shell, **(B)** state of digestion, **(C)** concentration of shells, **(D)** microbial inoculum, and **(E)** incubation time were optimized for their influence on deproteinization (protease), demineralization (calcium) and chitinolytic (GlcNAc) activity of *Streptomyces* S223 and S224. Lobster shell powder (LSP) devoid of microbial inoculum served as the negative control. Values represent mean ± SE (*n* = 3). Significant differences are indicated by different letters and asterisks (*P* < 0.05).

Each factor studied for optimizing the cultural conditions of the microorganisms holds a key role in achieving efficient degradation. There are two types of lobster shells, cooked and raw depending on the processing method and they differ in calcium, protein and chitin content. The state of digestion, solid vs. liquid, was used to study whether the microorganisms can perform degradation at low moisture content. The concentration of shells and load of microbial inoculum in the media were varied to investigate competent levels of both the factors for degradation in a particular time period. To determine the time required for maximum degradation, the incubation was carried over 4 weeks. The results of the optimization experiment are summarized in **Figure 2**. Protease activity was not detected in cooked shells because of low protein content in the shells. Deproteinization was not significantly (*P* = 0.96) affected by moisture level in the media. The protease units per mg protein were significantly (*P* < 0.0001) higher in 2% (w/v) concentration of shells than 5% concentration, whereas, microbial inoculum showed a significant (*P* < 0.0001) linear increase. Two weeks of incubation had higher protease units but it was not significantly (*P* = 0.06) different over the incubation period. A suggested explanation for decrease in protease units after 2 weeks could be the increased microbial proteins in the culture medium because the total protease units remained high but decreased when measured in terms of units per mg of protein. Cooked shell samples obtained from the S224 digestion had significantly (*P* = 0.001) higher calcium content. Increasing concentration of shells showed a significant (*P* < 0.0001) increase in calcium quantity and the volume of microbial inoculum had no significant (*P* = 0.72) effect on demineralization. Liquid digestion released more (*P* = 0.069) calcium into the media because of increased solubility. There was significantly (*P* < 0.0001) higher calcium content in the samples obtained on 7th day after incubation than at later time points and this might be due to the precipitation of calcium with organic acids.

Samples collected from raw shells and liquid digestion had significantly (*P* < 0.0001) higher GlcNAc content than cooked shells and solid-state digestion, respectively. Concentration of shells and microbial inoculum had linearly increased (*P* < 0.0001) the release of GlcNAc into the medium. GlcNAc quantified from 14 days of incubation was significantly (*P* = 0.0009) higher than other incubation periods. The optimization of time period for degradation of lobster shells is of particular interest because preliminary studies were not able to detect *N*-Acetylglucosamine in the samples collected 7 days after incubation with lobster shells (data not shown). Dimethylaminobenzaldehyde reagent binds to the *N*-Acetylglucosamine units at the terminal ends of broken chitin chains that resulted from mechanical grinding and hence samples from shells show *N*-Acetylglucosamine. The shells themselves did not have any chitinolytic activity and this was confirmed in the next experiment. Shrimp shells and chitin powder were reported to be excellent sources of carbon and nitrogen and assimilated by chitinolytic microorganisms (Wang et al., 2005; Xu et al., 2008). The similar chemical composition in lobster shells strongly induced microbial chitinase synthesis, where they must secrete both endochitinases and exochitinases to yield *N*-Acetylglucosamine as end product (Gooday, 1990). With lobster shells being used as the substrate carbon source, the rate of *N*-Acetylglucosamine production increased up to 2 weeks and then decreased over the incubation period. This suggested that the high concentration of the substrate and accessibility of all available sites of chitin particles for digestion by enzyme hydrolysis are the main reasons for the production of *N*-Acetylglucosamine during the initial stages. α-Chitin has antiparallel microfibril orientation with strong hydrogen bonding and it is the major form of chitin present in crustacean shells (Fabritius et al., 2009; Muzzarelli, 2009). There could be a limited accessibility to the β-glycosidic linkages in the interior chitin chains for the enzyme attack, and hence, the rate of chitinolysis being slowed down. Other researchers suggested the same reasons for the absence of chitin hydrolysis after 24 h incubation (Chen et al., 2010). The difference in the yield of *N*-Acetylglucosamine at later stages of incubation could be attributed to end product feedback inhibition, and/or enzyme denaturation during the reaction since intra or extracellular proteolytic enzymes could destroy chitinases of *Streptomyces* (Berger and Reynolds, 1958). Crude extracts were collected from the two *Streptomyces sp.* grown under these conditions. The results implied that 1% (v/v) microbe cultures of S233 and S224 efficiently degraded 1% (w/v) of raw lobster shells in M9 buffer (pH 7) incubated at 25°C, 150 rpm for 14 days. Crude extracts were collected from the two *Streptomyces sp.* grown under these conditions.

### Purification of Chitinase from Crude Extract

**FIGURE 3:**
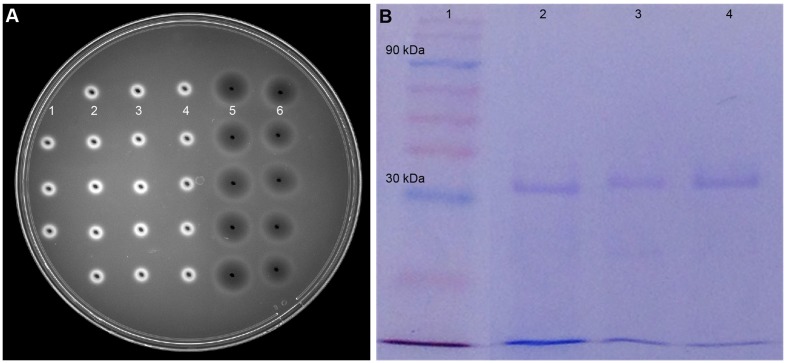
**Purification of chitinase enzyme by affinity chromatography.** **(A)** Dark circular zones indicate chitinase activity of microbe digested lobster shell extracts on glycol chitin agarose plates, visualized under UV transillumination. (1) is blank (water), (2) and (3) are culture filtrates of S223 and S224 grown on YEME, respectively, (4) is filtrate from undigested LSP, (5) and (6) are extracts of S223 and S224 grown on LSP, respectively. **(B)** SDS-PAGE analysis of purified chitinase from *Streptomyces sp.* S223. Lanes (1) Molecular weight markers 10–175 kDa (2) Chitinase from *Streptomyces griseus* (3) Purified chitinase from S223 after affinity chromatography (4) S223 chitinase concentrated by Amicon^®^ PM30 filter unit.

Gel diffusion assay using glycol chitin was effective to detect chitinase in the microbial culture extracts prior to enzyme purification (Supplementary Figure S6). The dark circular zones indicated that two *Streptomyces* species isolated from soil samples produce chitinases in lobster shell media (**Figure 3A**). Culture extracts obtained from LSP exhibited chitinase activity whereas extracts from YEME grown cultures did not show any chitinase activity. This proved that chitinase synthesis of *Streptomyces* is strongly dependent on the substrate present and several chitinase genes, *chiA*, *chiB*, *chiC*, *chiD*, and *chiF* of *S. coelicolor* A3 were found to be transcribed in the presence of chitin but down regulated in the presence of glucose (Saito et al., 2000). Cell-free enzyme preparations of from *Streptomyces* have been reported to digest chitin (Reynolds, 1954) and the predominant enzyme secreted by *S. griseus* was chitobiosidase (Berger and Reynolds, 1958), whereas in S223 endochitinase and β-N-Acetylglucosaminidase were abundant. Chitinase obtained from S223 purified by affinity chromatography resulted in 3.2-fold purification at the final stage (**Table 1**) and its molecular weight was found to be ∼30kDa (**Figure 3B**). Brzezinska et al. (2013) purified chitinase up to 3.9-fold from *S. albidoflavus* and Mukherjee and Sen (2006) obtained a 3.19-fold purified chitinase from *S. venezulae* P10. Many researches have reported the molecular weight of purified chitinase from *Streptomyces* species was between 20 and 71 kDa (Joo, 2005; Han et al., 2009). Purification of chitinase from S224 remained a challenge in spite of 45.6 units chitobiosidase/mg of protein in the crude extract (data not shown).

**Table 1:** Chitinase activity of S223 culture filtrate using different substrates.

Purification step	Specific activity (Units/mg protein)				Purification factor
		
	Endo chitinase	Chitobio sidase	N-Acetyl β–glucosaminidase	Total	
Culture supernatant	23.05	8.18	27.88	59.11	1
NH_4_SO_4_ (85%) dialysis	24.43	10.05	36.47	70.96	1.2
Affinity adsorption	49.84	38.31	58.81	146.96	2.4
Amicon^®^ PM30	66.68	52.93	73.1	192.71	3.2


### Microbe Digested Lobster Shell Extracts Exert Antimicrobial Activity

**FIGURE 4:**
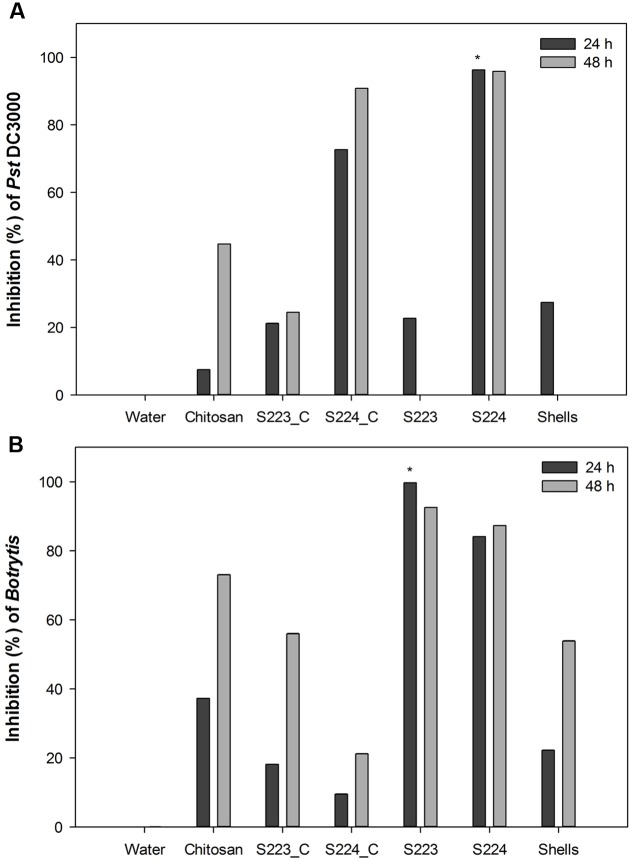
**Antimicrobial activity of lobster shell digested extracts on pathogen growth.** Inhibition percentage of **(A)** *Pst* DC3000 and **(B)** *Botrytis cinerea* was calculated respective to control treatment. Water – control, Chitosan – positive control, S223_C, S224_C – control extracts of S223 and S224 grown in YEME, S223 – extract of S223 digested lobster shells, S224 – extract of S224 digested lobster shells, Shells – extract of undigested lobster shells. Values represent mean ± SE (*n* = 6). Asterisks indicate significant difference (*P* < 0.05).

The growth of bacterial pathogen, *P. syringae* pv. *tomato* DC3000 was significantly (*P* < 0.0001) inhibited by the addition of extracts obtained from S224 digested LSP (**Figure 4A**). Similar antimicrobial activity of chitosan on *P. syringae* and *S. scabies* (Vruggink, 1970; Ferrante and Scortichini, 2010). Spore germination and hyphal growth of *B. cinerea* was significantly inhibited (*P* = 0.0028) by the addition of S223 digested LSP extract (**Figure 4B** and Supplementary Figure S7). S224 digested LSP extract showed at par reduction in the growth of *B. cinerea*. In addition to the antibacterial activity, chitosan showed fungistatic activity against both biotrophic and necrotrophic pathogens (Sharp, 2013). The antimicrobial activity of the microbial digested lobster shell extracts suggested presence of chitin derived and chitosan like compounds. Xu et al. (2007) reported that chitosan application was effective to control post-harvest decay in grapes caused by *B. cinerea*.

Culture filtrates from S223 and S224 grown on YEME inhibited pathogen growth but the activity was lower than that exhibited in lobster shell digested extracts. The higher inhibitory activity observed in lobster shell digested extracts implied that chitinase production is specific to the media. Chater (2006) reported the production of secondary metabolites by *Streptomyces*, which had antibiotic properties and the secretion was regulated by extracellular signaling molecules. The antimicrobial property of the extracts is predominantly due to the chitinases present. When chitin in fungal cell wall is digested by chitinases, the associated β-glucan is solubilized as well causing the disruption of the cell wall (Schlumbaum et al., 1986). Chitinases also possess lysozyme properties and they digest peptidoglycan of the bacterial cell walls, which is made of alternating β-1,4 linked residues of *N*-acetylglucosamine and *N*-acetylmuramic acid resembling the structure of chitin (Roberts and Selitrennikoff, 1988). The filtrates obtained from lobster shell digestion and YEME might also contain secondary metabolites possessing antibiotic properties that had detrimental effect on the growth of plant pathogens. Extracts from undigested lobster shells also exhibited 27% inhibition of *Pst* DC3000 at 24 h but showed 50–55% inhibition of *Botrytis* at 48 h after incubation. This suggested that chitin itself has certain extent of antimicrobial properties (Muzzarelli, 2009; Xia et al., 2011).

### Reduction of Disease Severity in Arabidopsis

**FIGURE 5:**
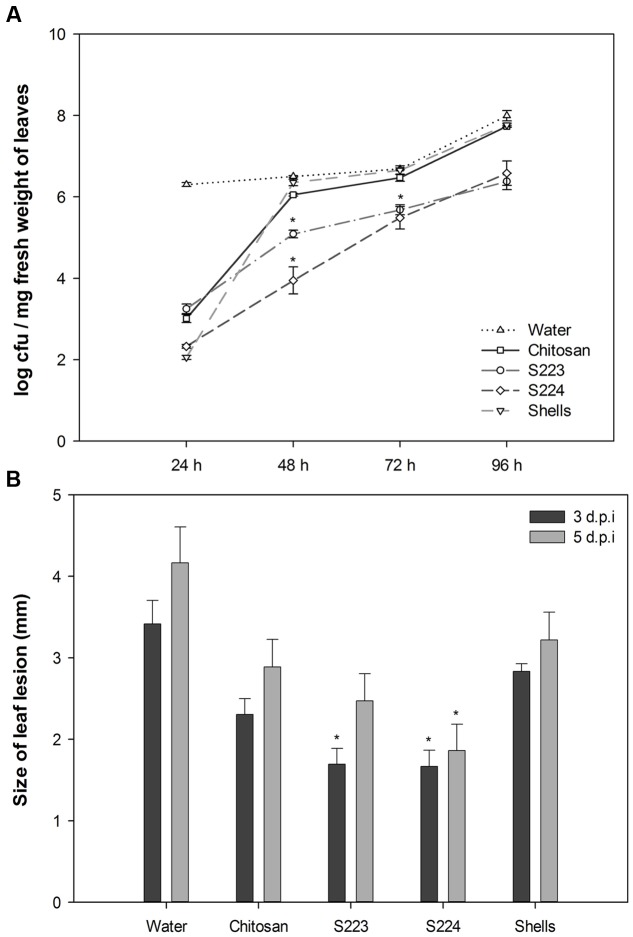
**Phenotypic observation of disease incidence in Arabidopsis leaves.** **(A)** Proliferation of bacteria in leaves harvested at 24, 48, 72, and 96 h post-inoculation was enumerated by colony forming units (cfu) of *Pst* DC3000 on Kings B agar plates. Values represent mean ± SE of three independent samples pooled from nine randomly selected plants (*n* = 3). **(B)** Size of leaf lesion measured on 3rd and 5th day post-inoculation of *Botrytis* on six leaves per plant. Plants were treated with Water – control, Chitosan – positive control, S223 – extract of lobster shells digested by S223, S224 – extract of lobster shells digested by S224, Shells – extract of undigested lobster shells. Values represent mean ± SE (*n* = 6). Asterisks indicate significant difference (*P* < 0.05).

Soil amendment by chitosan is widely used to control fungal, bacterial and viral diseases in numerous crops (Sharp, 2013) and therefore used as a positive control in disease experiments. Arabidopsis leaves infected with *Pst* DC3000 showed yellowing symptoms on 3rd day after inoculation and visual observations showed that plants treated with microbe digested lobster shell extracts strongly restricted disease spread compared to other treatments (Supplementary Figures S8, S9). *Pst* DC3000 colonizing Arabidopsis leaves were enumerated on Kings B agar plates and S223 and S224 digested lobster shell extracts showed a significant (*P* < 0.0001) reduction of number of cfu during the infection period (**Figure 5A**). *Botrytis* infection resulted in rapidly expanding, water-soaked lesions formed at the spot of inoculation and were measured on 3rd and 5th day after inoculation. Digested lobster shell extracts showed significant (*P* < 0.0001) reduction on the spread of leaf lesion (**Figure 5B**). The infected spots have become localized necrosis resulting from increased disease resistance in plants treated with microbe digested lobster shell extracts (Supplementary Figure S10). Chitosan treated plants showed less disease incidence when compared to water treated (control) plants. Phenotypic observation of disease resistance upon application of the extracts is due to the combined effect of antibiotic properties and elicitation of plant defense responses against *Pst* DC3000 and *B. cinerea*.

### Lobster Shell Extracts Induce Disease Resistance in Arabidopsis

**FIGURE 6:**
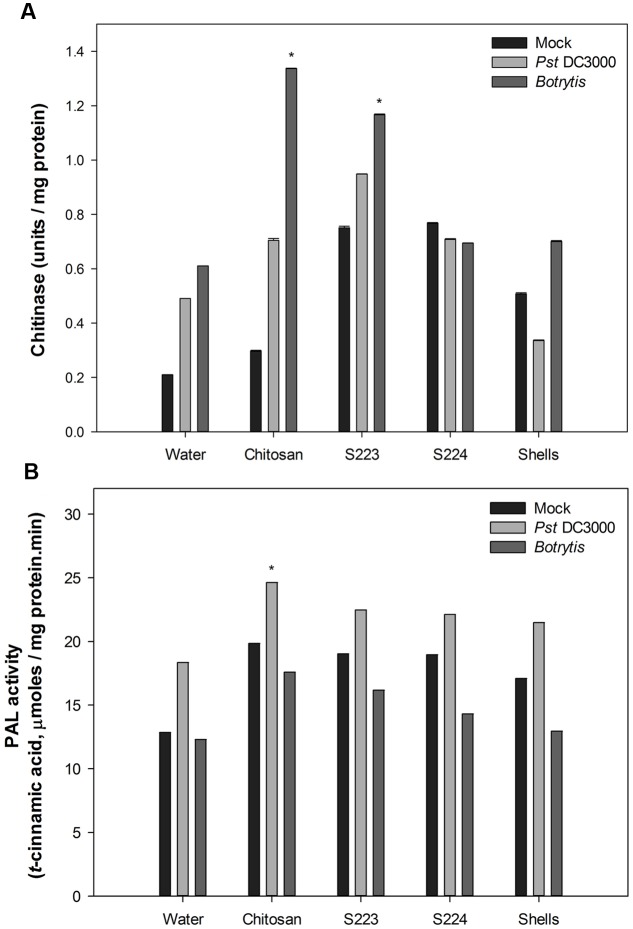
**Enzyme activity in Arabidopsis leaf tissues.** **(A)** Chitinase and **(B)** Phenylalanine ammonia lyase activity quantified in Mock, *Pst* DC3000 and *Botrytis* infected leaf tissues harvested at 48 h post-treatment or inoculation. Plants were treated with Water – control, Chitosan – positive control, S223 – extract of lobster shells digested by S223, S224 – extract of lobster shells digested by S224, Shells – extract of undigested lobster shells. Values represent mean ± SE of three independent samples pooled from nine randomly selected plants (*n* = 3). Asterisks indicate significant difference (*P* < 0.05).

The function of digested lobster extracts as elicitors of defense responses in Arabidopsis were determined by tissue biochemical and gene expression analysis. Mechanisms of ISR and SAR in plants are mediated by salicylic acid (SA), jasmonic acid (JA), and ethylene (ET) dependent pathways, which regulate downstream transcription of genes associated with synthesis of pathogenesis related (PR) proteins and phytoalexins in disease resistant phenotypes (Grant and Lamb, 2006; Jones and Dangl, 2006; Spoel and Dong, 2012). Plant chitinases are well characterized for their role as PR proteins (Collinge et al., 1992; Busam et al., 1997). Phenylalanine ammonia lyase (PAL) catalyzes the first committed step of phenylpropanoid pathway that synthesizes SA (Coquoz et al., 1998). Quantification of chitinase and PAL are used in the present study as induced resistance markers of biotic stress and their increased activity in the infected plants is positively linked with increased disease resistance (Summermatter et al., 1995). Chitinase activity was not detected in measurable quantity in the Arabidopsis leaf tissues after 24 h of treatment or inoculation with pathogens. Chitosan treated plants exhibited significantly (*P* < 0.0001) higher chitinase activity in the leaf tissue after 48 h upon infection with *Botrytis* (**Figure 6A**). S223 extracts had strongly induced chitinase synthesis in place of both *Pst* DC3000 (0.95 U mg^-1^protein) and *Botrytis* (1.33 U mg^-1^protein) infection. The reduced chitinase activity in S224 treated plants suggested that it might induced other defense mechanisms. PAL activity is an important indicator of stress conditions and fungal infection (Huang et al., 2010). Application of S223 and S224 digested lobster shell extracts showed significant (*P* = 0.0044) difference in PAL activity between treatments after 48 h (**Figure 6B**). When compared to mock and *Botrytis* infection, PAL activity was significantly (*P* < 0.0001) higher in plants with *Pst* DC3000 at 48 h post-infection. Moreover, chitosan treated plants (24.6 μmol) showed increase in PAL activity than lobster shell extracts (∼22 μmol) treated plants. The lobster shell extracts, both digested and undigested, had elicited chitinase and PAL activity in non-infected (mock) plants, which suggested their possible functions as an elicitor of PAMPs.

**FIGURE 7:**
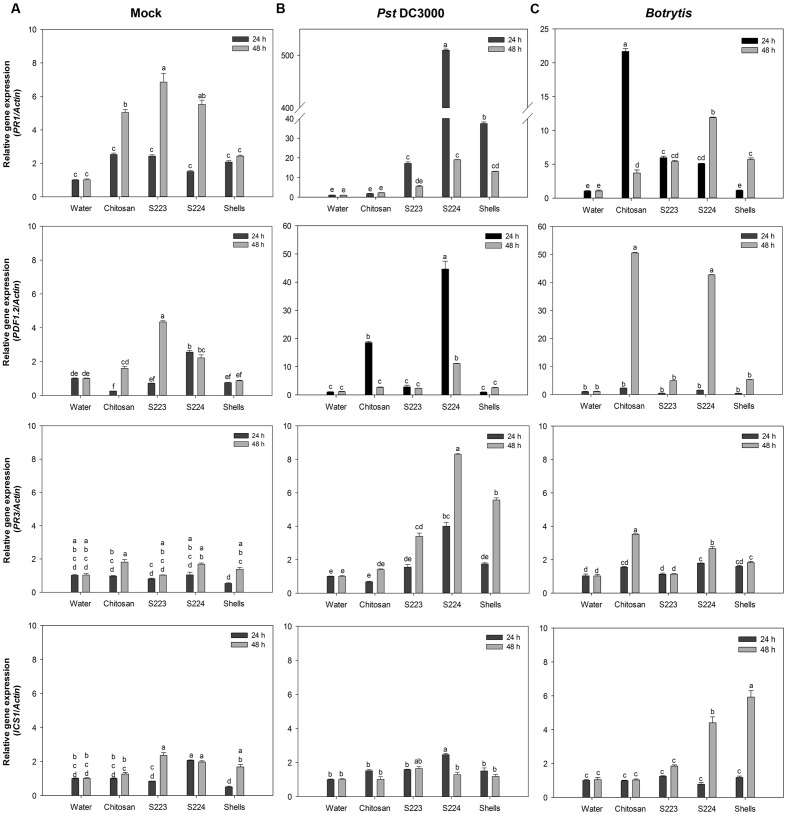
**Gene expression in Arabidopsis leaf tissues.** Relative expression levels of *PR1*, *PDF1.2*, *PR3*, *ICS1* in **(A)** Mock, **(B)** *Pst* DC3000 and **(C)** *Botrytis* infected leaf tissues harvested at 24 and 48 h post-treatment or inoculation. Plants were treated with Water – control, Chitosan – positive control, S223 – extract of lobster shells digested by S223, S224 – extract of lobster shells digested by S224, Shells – extract of undigested lobster shells. Values represent mean ± standard error of three independent samples pooled from nine randomly selected plants (*n* = 3). Significant differences are indicated by different letters (*P* < 0.05).

The most commonly studied genes that are regulated by the signaling pathways include *PR1*, *PR3*, *PDF1*.2, and *ICS1*. Their expression levels were higher in infected than non-infected (mock) plants and different at 24 and 48 h after treatment. The interaction between the treatments and time had a significant (*P* < 0.0001) effect on the transcript abundance of the genes (**Figure 7**). The expression of *PR1* and *PDF1*.2 were greater in *Pst* DC3000 and *Botrytis* infected plants, respectively. S224 extract treatment resulted in the highest (*P* < 0.0001) expression of *PR1* (500-fold) in plants infected with *Pst* DC3000 at 24 h. Plants treated with chitosan (∼22-fold) showed increase in PR1 expression upon *Botrytis* at 48 h post-infection (*P* < 0.0001). JA dependent plant defensin, *PDF1*.2 expression was significantly higher in S224 treated plants (∼45-fold) infected with *Pst* DC3000 at 24 h (*P* < 0.0001). The expression of *PDF1*.2 was strongly induced in plants treated with chitosan (50-fold) and S224 extracts (>40-fold) under *Botrytis* infection at 48 h (*P* < 0.0001). The expression of *PR3* was significantly increased at 48 h in S224 extract treated plants (8-fold) after infection with *Pst* DC3000 (*P* = 0.0001) and chitosan treated plants (3.5-fold) followed by S224 extract under *Botrytis* infection (*P* < 0.0001). The expression of Isochorismate synthase, *ICS1* was higher at 24 h in S224 treated (2.5-fold) – *Pst* DC3000 infected plants (*P* = 0.01) and 48 h in shells (6-fold) and S224 (4.4-fold) treated – *Botrytis* infected plants (*P* < 0.0001). A general trend observed was increase in gene expression at 24 h with *Pst* DC3000 infection whereas, under *Botrytis* infection the expression was higher at 48 h.

The upregulation of *PR1* in plants suggest the onset of SAR mediated by SA pathway since Arabidopsis mutants unable to accumulate SA have shown reduced expression levels of *PR1* (Glazebrook, 2001). *Botrytis*, a necrotrophic pathogen, induce SA mediated SAR (Murphy et al., 2000; Audenaert et al., 2002). The increased PAL activity in chitosan treated plants correlated to the expression of *PR1* at 48 h after infection. Isochorismate synthase 1 (*ICS1*) has been shown to be necessary for SA synthesis pathway that is independent of PAL (Wildermuth et al., 2001) and its expression again relates to *PR1* induction by *ICS1* mediated SA pathway. Interestingly, the levels of *ICS1* were remarkably higher in *Botrytis* infected than mock and *Pst* DC3000 plants. Induction of *PDF1.2* suggested the up-regulation of JA mediated defense pathways, because among the plant defensins only *PDF1.2* was induced upon pathogen challenge (Thomma et al., 2002). Subramanian et al. (2011) showed that *PDF1.2* was induced in Arabidopsis infected with *Pst* DC3000. *Botrytis* also caused the induction of JA mediated *PDF1.2*, which regulates an antifungal defensin like peptide (Penninckx et al., 1998; Zimmerli et al., 2001). *PR3* gene encodes for endochitinase and is regulated by ET/JA dependent pathway (Glazebrook, 2001) and *PR3* expression indicated endochitinase activities being induced in the plants. Expression of some chitinase genes is attenuated in the presence of SA or JA suggesting an alternate signaling pathway in the defense response (Shinya et al., 2007). The undigested lobster shell extract treated plants also exhibited an increase in enzyme activity and gene expression levels due to the presence of chitinous substances, which acted as PAMPs (Eckardt, 2008). These results confirmed that disease resistance was induced in the plants upon treatment with lobster shell extracts and different signaling pathways activated the defense responses. The chitin and its derivatives obtained after digestion of lobster shell by S223 and S224 induced resistance against *P. syringae* and *B. cinerea* in *Arabidopsis* by increased enzyme activity and expression of defense responsive genes.

## Conclusion

The optimization of lobster shell degradation by two *Streptomyces sp*., S223 and S224 and elucidation of the chitinolytic system offers a precognition for an industrial scale process to extract chitin derivatives from the shells. Metabolic profiling of the culture extracts will help to identify the compounds that act as elicitors, which are often recognized by the plants as PAMPs (Zipfel, 2009). In order to gain a better understanding of the elicitation of plant defense mechanisms by the lobster shell extracts, several genetic and biochemical measures of disease resistance in plants across different taxonomical groups and agronomical importance, under a range of environment and resource conditions are required. The digested lobster shell extracts induced disease resistance in plants through elicitation of defense signaling mechanisms that were accelerated when exposed to pathogen infection. Their antimicrobial property further enhanced plant protection, which rationalize their application as an environmentally safe alternative to chemical controls used in plant protection. This study provides new insights that can contribute to alternative methods of recycling lobster shell waste and its potential utilization in the future.

## Author Contributions

BP conceived the idea. GI, SR, GS, SA, PP, and BP designed experiment and analyzed data. GI and SR performed experiments. GI, SR, PS, and BP interpreted the data and prepared the manuscript.

## Conflict of Interest Statement

The authors declare that the research was conducted in the absence of any commercial or financial relationships that could be construed as a potential conflict of interest.
