# Supplementary material for: Microbial Degradation of Lobster Shells to Extract Chitin Derivatives for Plant Disease Management
Source: Front Microbiol. 2017 May 5;8:781. doi: 10.3389/fmicb.2017.00781 (PMC5418339; doi:10.3389/fmicb.2017.00781)
Supplement: Supplementary file 1 [file Table_1.DOCX]

| **Supplementary Table S1**.  Gene specific primers used to study induced disease resistance in Arabidopsis | | |
| --- | --- | --- |
| Gene | Gene locus | Gene specific primers (Invitrogen™) |
| *PR1* | AT2G14610.1 | F 5’-ACATGTGGGTTAGCGAGAAG-3’  R 5’-ACTTTGGCACATCCGAGTCT-3’ |
| *PR3* | AT3G12500.1 | F 5’-ACGAAGGATCTTTGGTTGTA-3’  R 5’-ACATCATTAACGGTGGATTG-3’ |
| *PDF1.2* | AT5G44420.1 | F 5’-TGCTGGGAAGACATAGTTGC-3’  R 5’-TGGTGGAAGCACAGAAGTTG-3’ |
| *ICS1* | AT1G74710.1 | F 5’-TTCTTCCGTGACCTTGATGG-3’  R 5’-CCAAAAGGTTCCCATTCAAC-3’ |
