# Supplementary material for: Microbial Degradation of Lobster Shells to Extract Chitin Derivatives for Plant Disease Management
Source: Front Microbiol. 2017 May 5;8:781. doi: 10.3389/fmicb.2017.00781 (PMC5418339; doi:10.3389/fmicb.2017.00781)
Supplement: Supplementary file 2 [file Presentation_1.PPTX]

## Slide 1
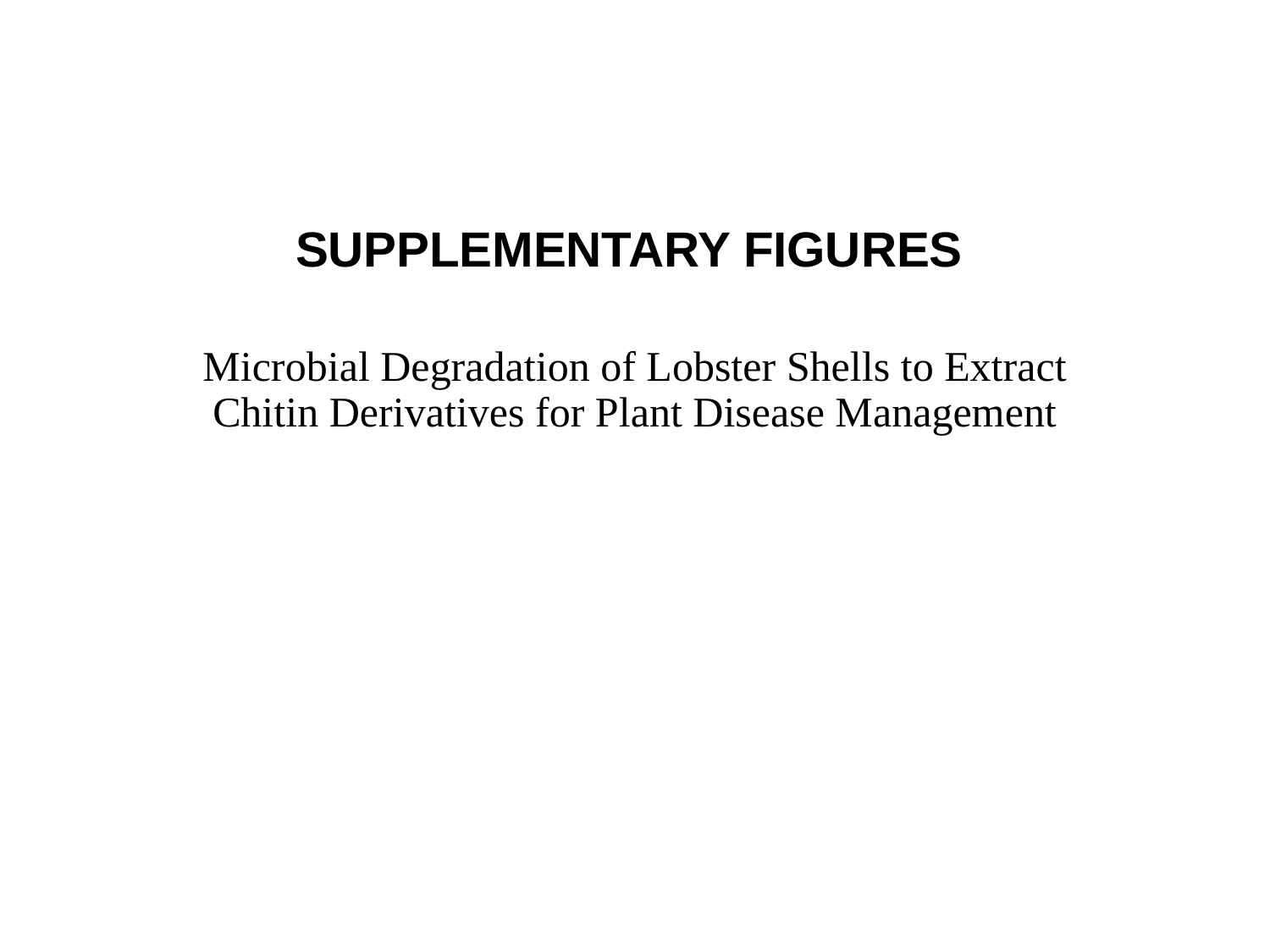

# SUPPLEMENTARY FIGURES
Microbial Degradation of Lobster Shells to Extract Chitin Derivatives for Plant Disease Management

## Slide 2
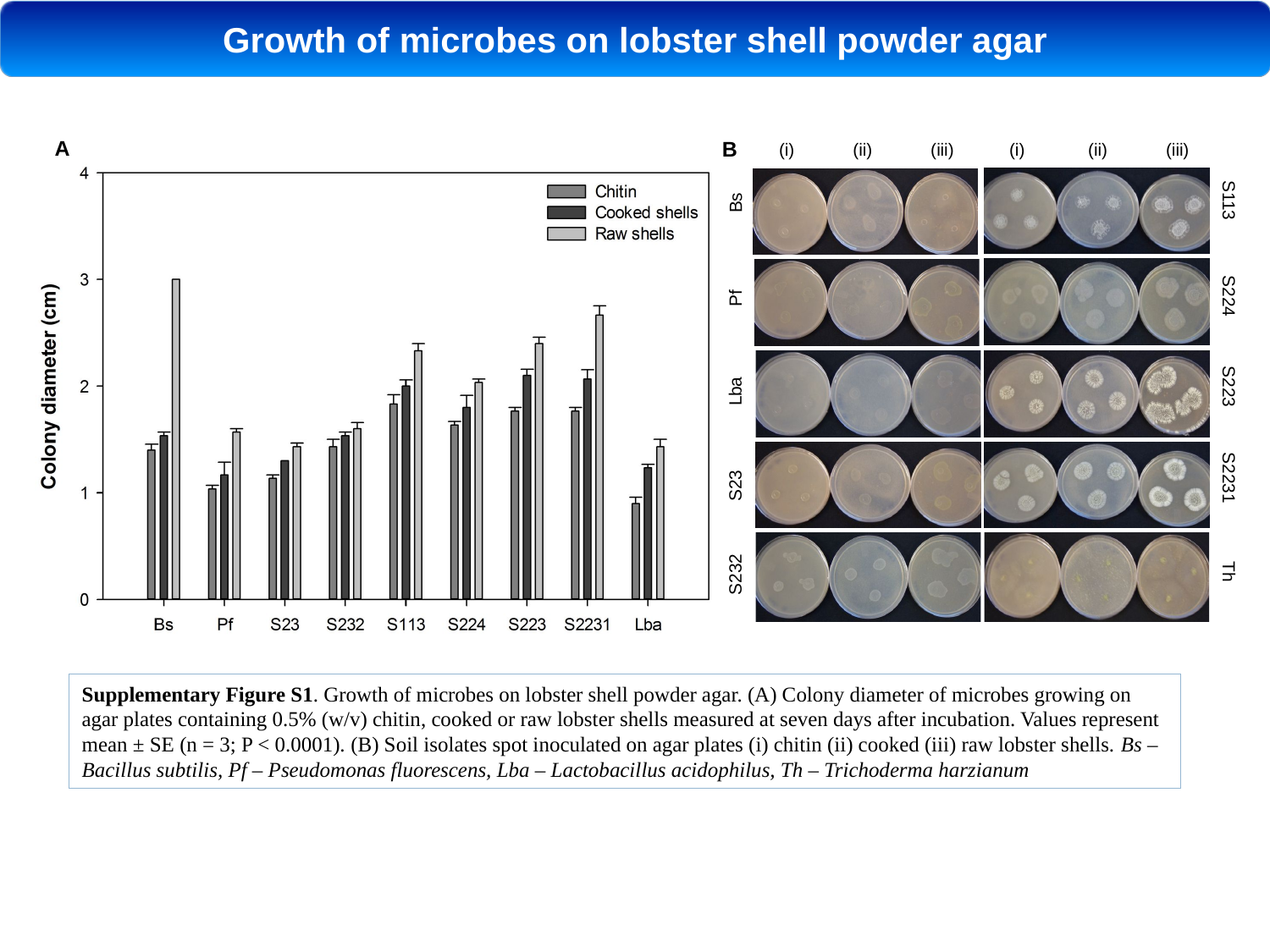

Growth of microbes on lobster shell powder agar
A
B
 (i) (ii) (iii)
 (i) (ii) (iii)
S113
Bs
S224
Pf
S223
Lba
S2231
S23
Th
S232
Supplementary Figure S1. Growth of microbes on lobster shell powder agar. (A) Colony diameter of microbes growing on agar plates containing 0.5% (w/v) chitin, cooked or raw lobster shells measured at seven days after incubation. Values represent mean ± SE (n = 3; P < 0.0001). (B) Soil isolates spot inoculated on agar plates (i) chitin (ii) cooked (iii) raw lobster shells. Bs – Bacillus subtilis, Pf – Pseudomonas fluorescens, Lba – Lactobacillus acidophilus, Th – Trichoderma harzianum

## Slide 3
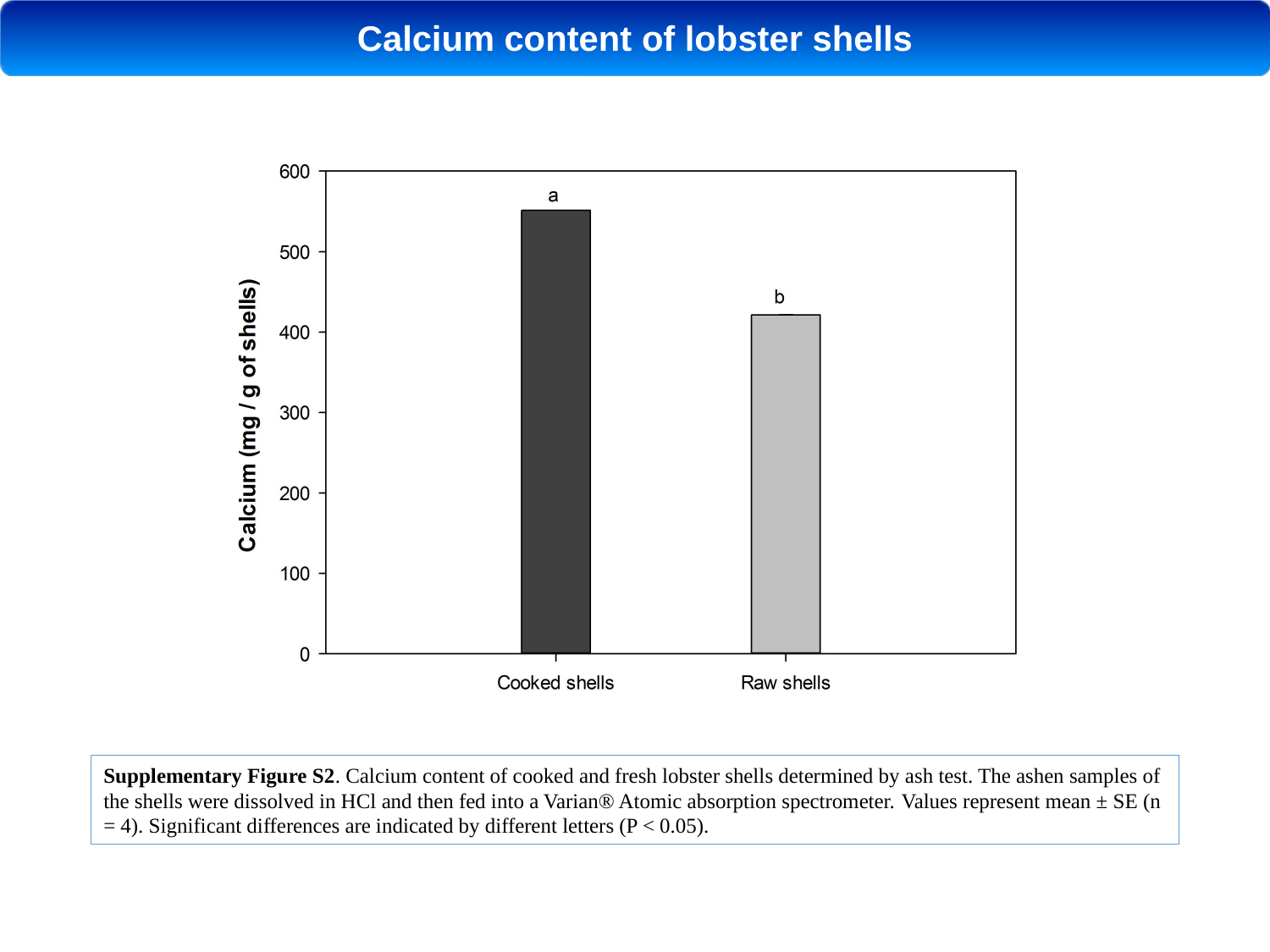

Calcium content of lobster shells
Supplementary Figure S2. Calcium content of cooked and fresh lobster shells determined by ash test. The ashen samples of the shells were dissolved in HCl and then fed into a Varian® Atomic absorption spectrometer. Values represent mean ± SE (n = 4). Significant differences are indicated by different letters (P < 0.05).

## Slide 4
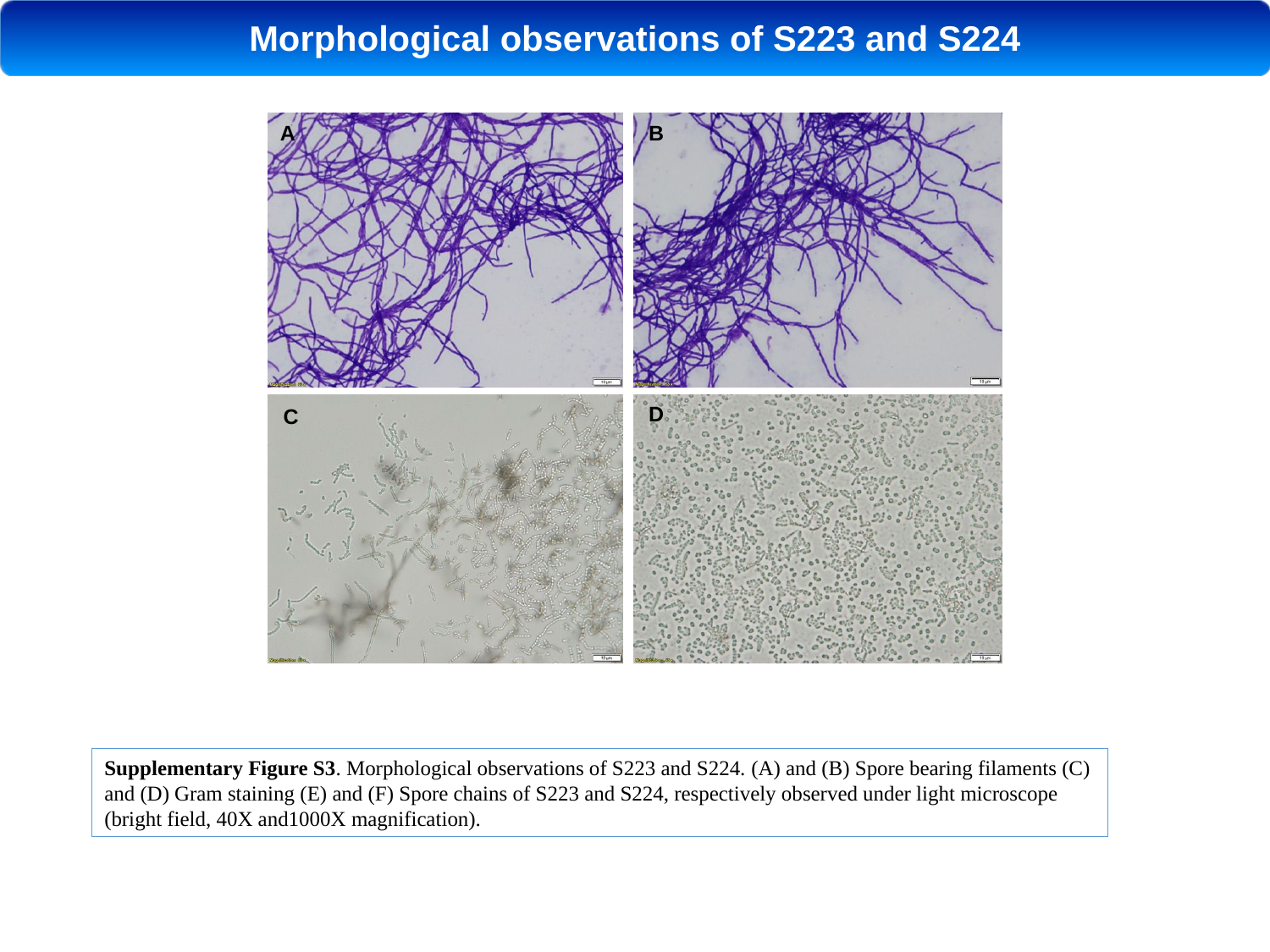

Morphological observations of S223 and S224
A
B
D
C
Supplementary Figure S3. Morphological observations of S223 and S224. (A) and (B) Spore bearing filaments (C) and (D) Gram staining (E) and (F) Spore chains of S223 and S224, respectively observed under light microscope (bright field, 40X and1000X magnification).

## Slide 5
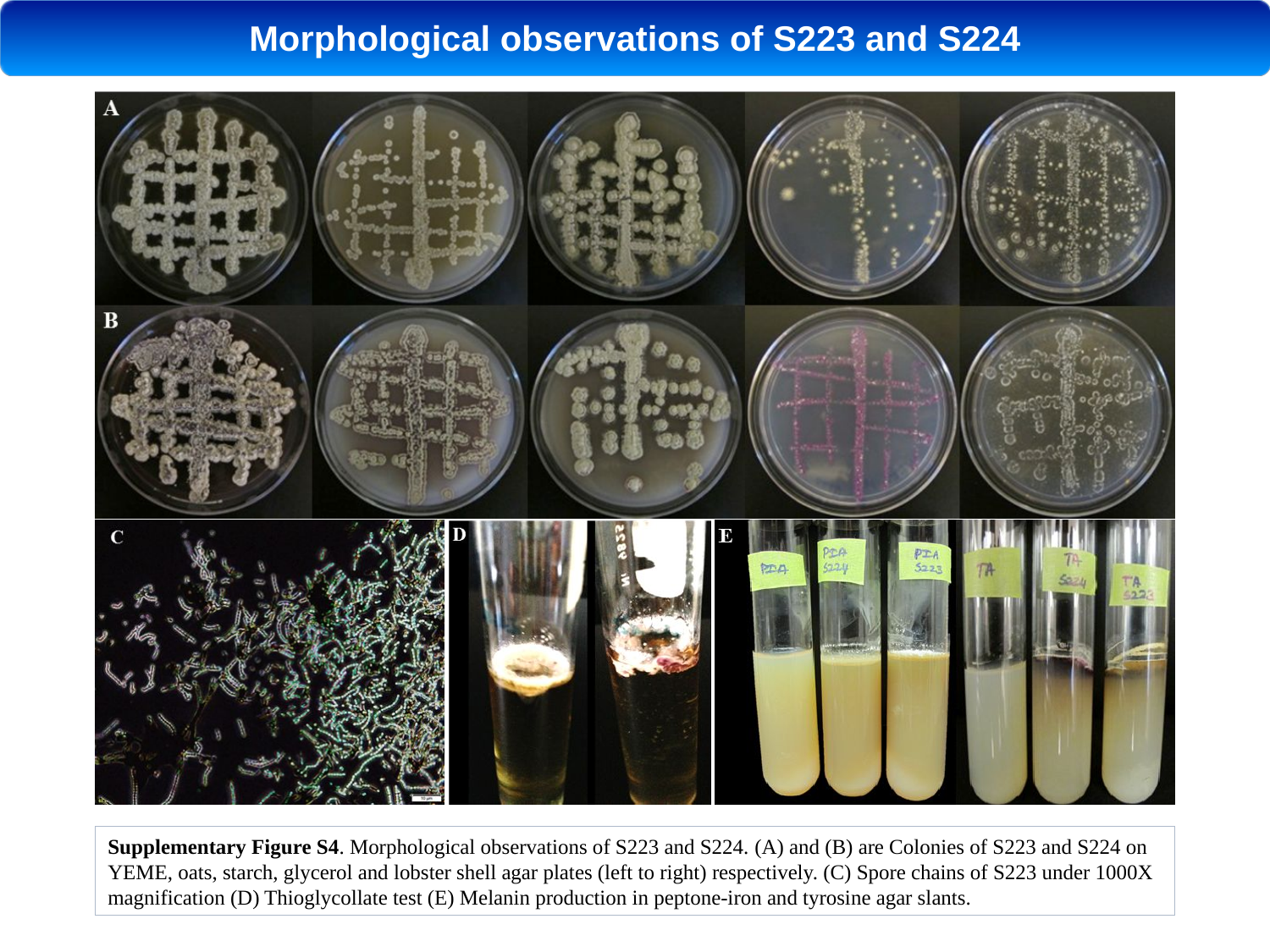

Morphological observations of S223 and S224
Supplementary Figure S4. Morphological observations of S223 and S224. (A) and (B) are Colonies of S223 and S224 on YEME, oats, starch, glycerol and lobster shell agar plates (left to right) respectively. (C) Spore chains of S223 under 1000X magnification (D) Thioglycollate test (E) Melanin production in peptone-iron and tyrosine agar slants.

## Slide 6
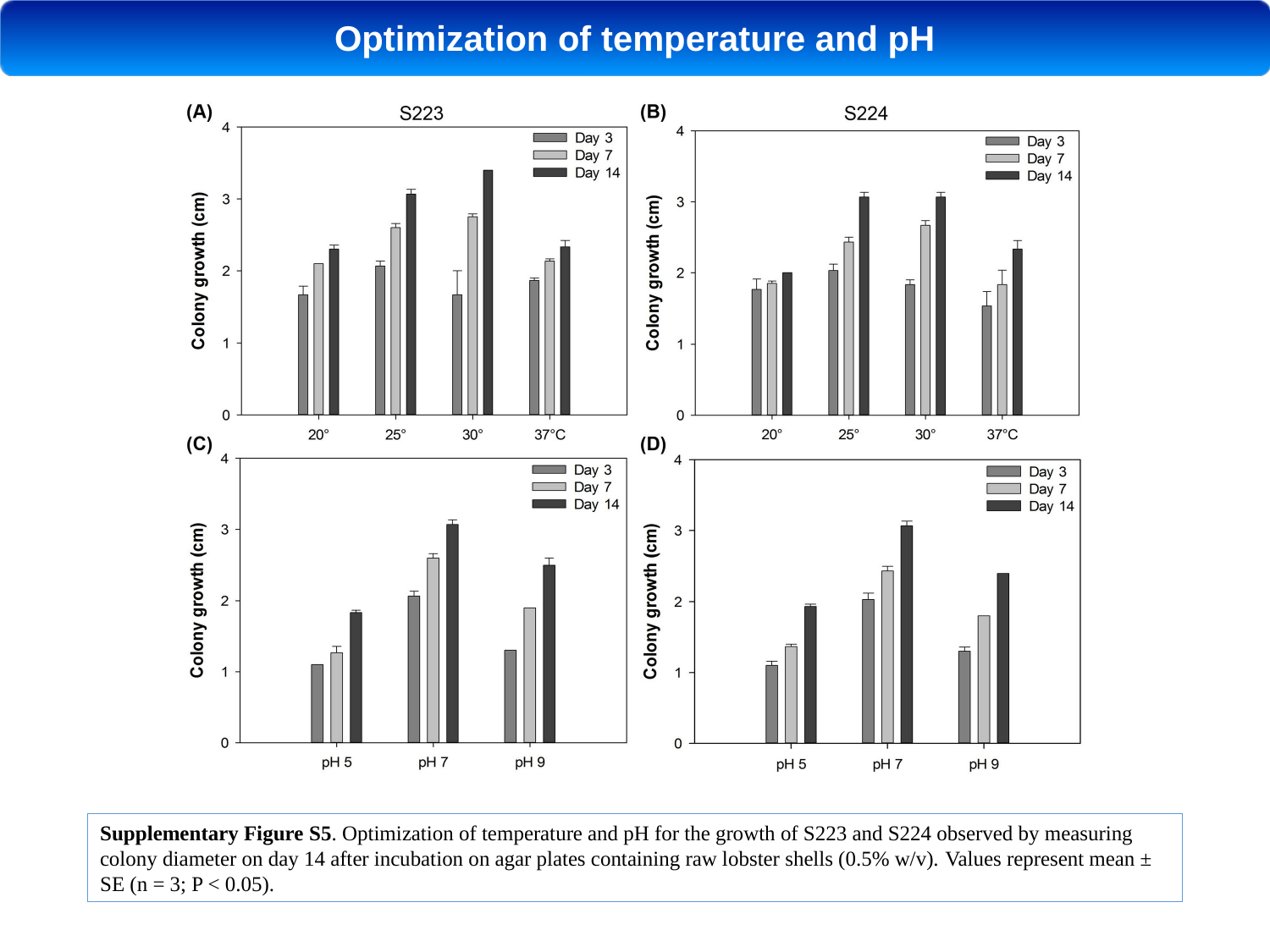

Optimization of temperature and pH
Supplementary Figure S5. Optimization of temperature and pH for the growth of S223 and S224 observed by measuring colony diameter on day 14 after incubation on agar plates containing raw lobster shells (0.5% w/v). Values represent mean ± SE (n = 3; P < 0.05).

## Slide 7
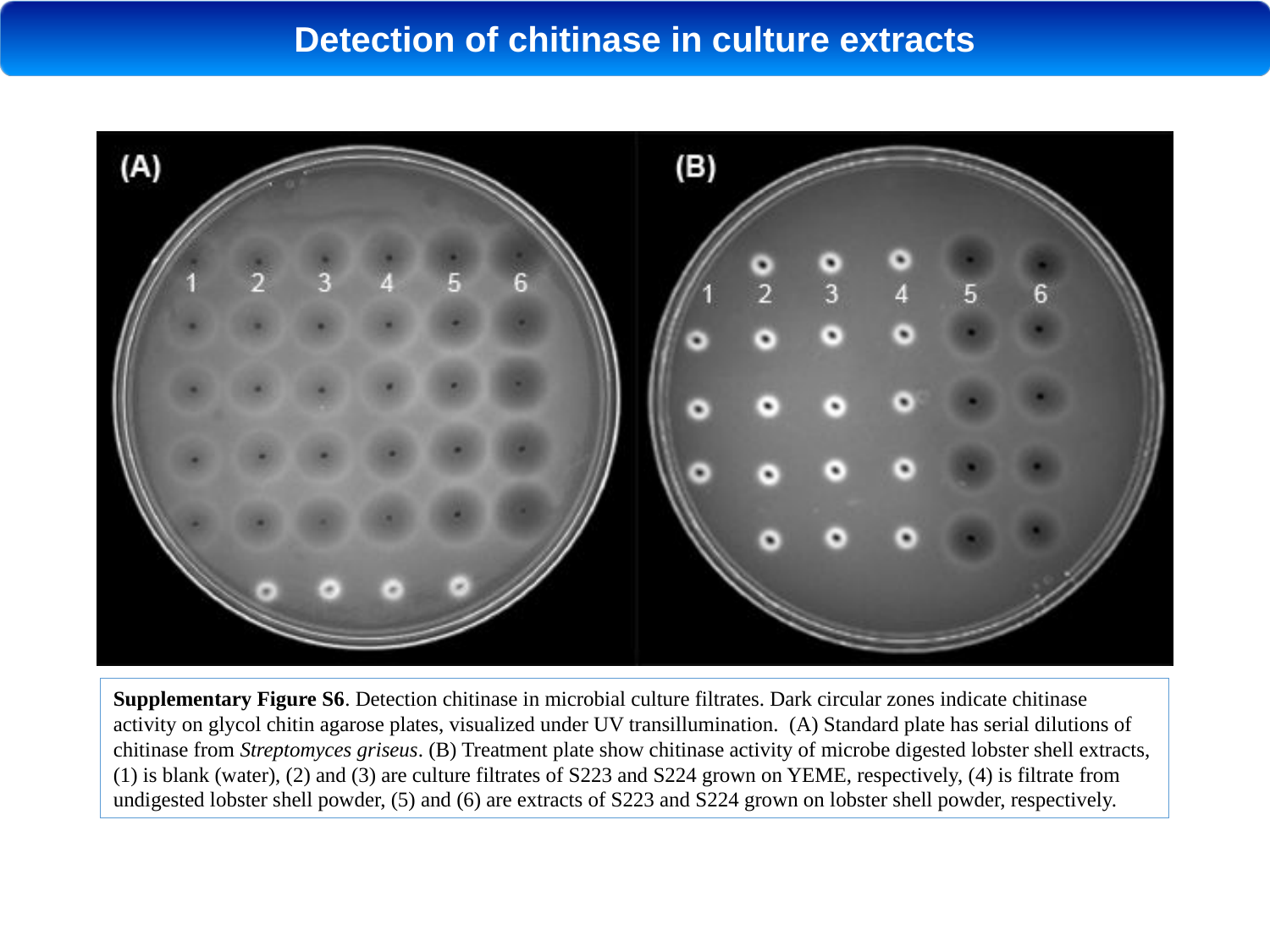

Detection of chitinase in culture extracts
Supplementary Figure S6. Detection chitinase in microbial culture filtrates. Dark circular zones indicate chitinase activity on glycol chitin agarose plates, visualized under UV transillumination. (A) Standard plate has serial dilutions of chitinase from Streptomyces griseus. (B) Treatment plate show chitinase activity of microbe digested lobster shell extracts, (1) is blank (water), (2) and (3) are culture filtrates of S223 and S224 grown on YEME, respectively, (4) is filtrate from undigested lobster shell powder, (5) and (6) are extracts of S223 and S224 grown on lobster shell powder, respectively.

## Slide 8
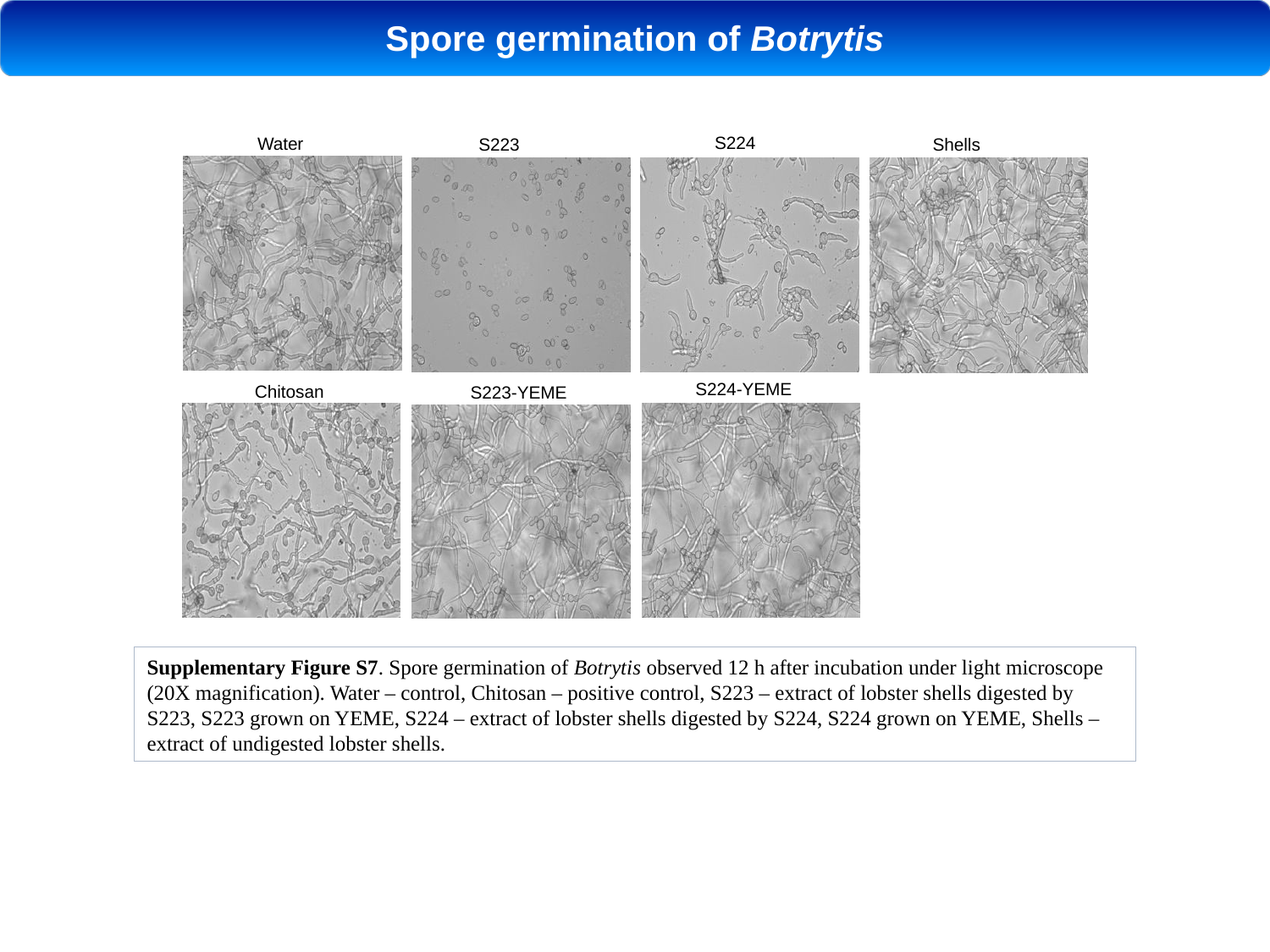

Spore germination of Botrytis
S224
Water
S223
Shells
S224-YEME
Chitosan
S223-YEME
Supplementary Figure S7. Spore germination of Botrytis observed 12 h after incubation under light microscope (20X magnification). Water – control, Chitosan – positive control, S223 – extract of lobster shells digested by S223, S223 grown on YEME, S224 – extract of lobster shells digested by S224, S224 grown on YEME, Shells – extract of undigested lobster shells.

## Slide 9
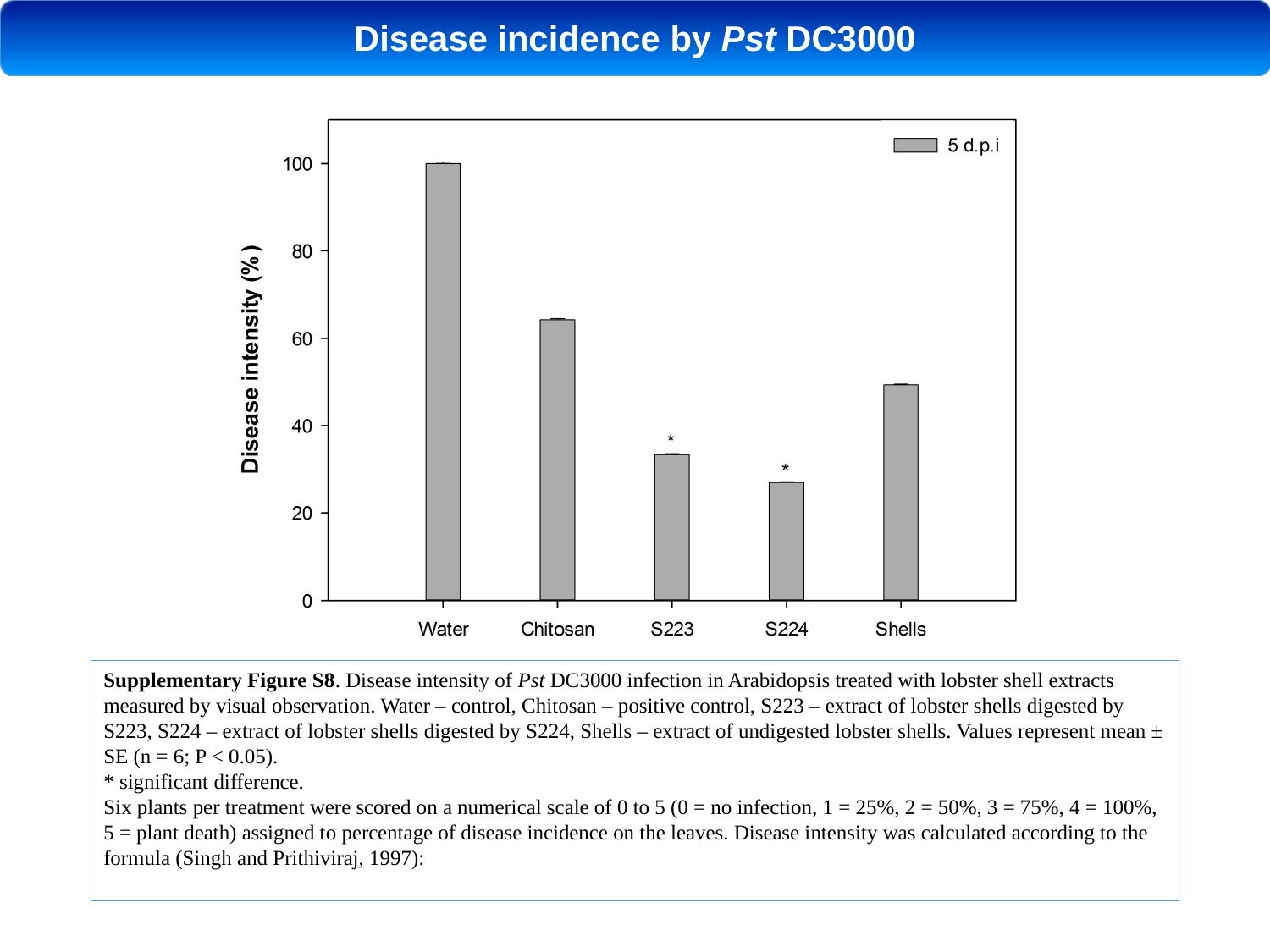

Disease incidence by Pst DC3000

## Slide 10
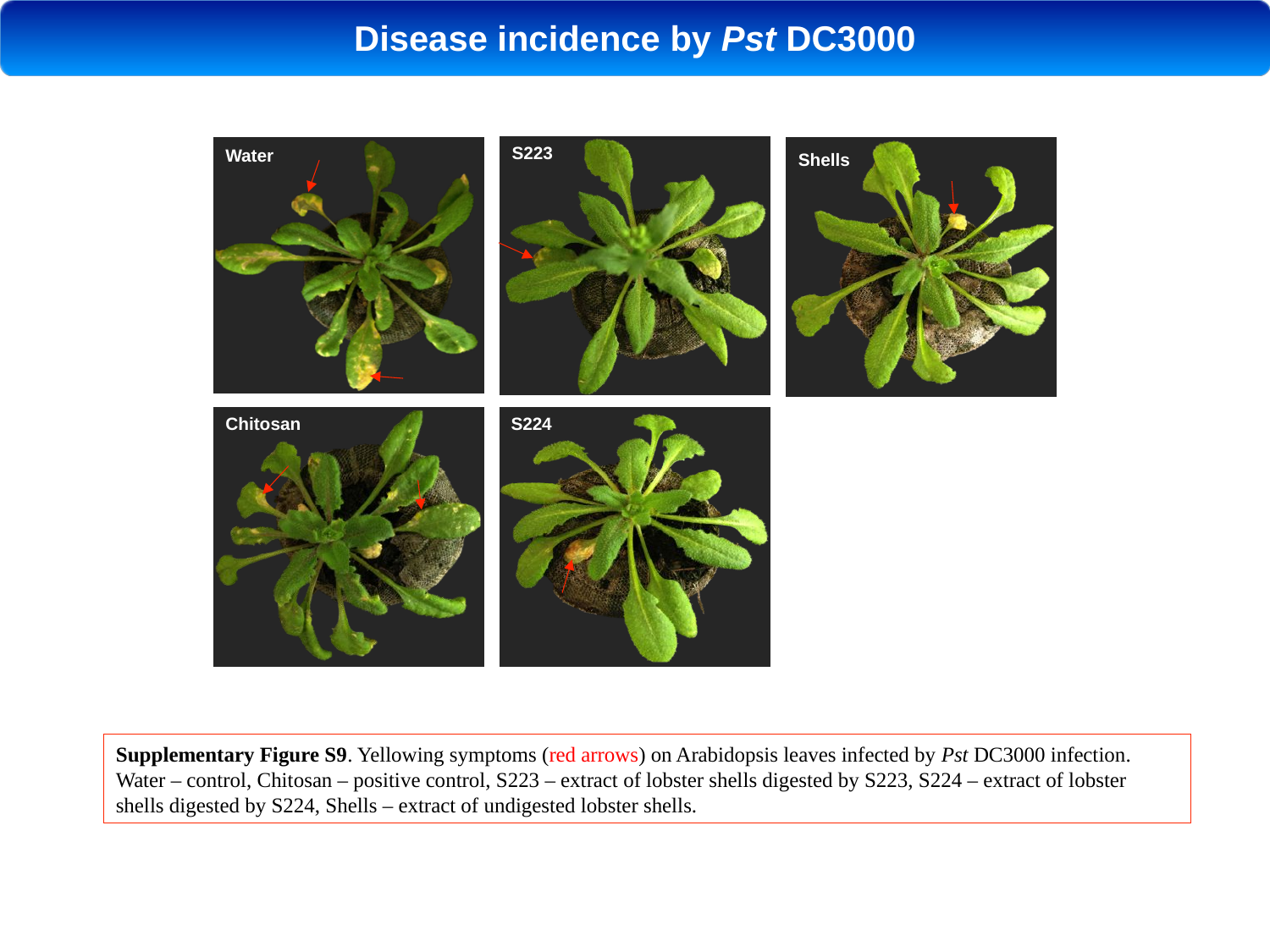

Disease incidence by Pst DC3000
S223
Water
Shells
Chitosan
S224
Supplementary Figure S9. Yellowing symptoms (red arrows) on Arabidopsis leaves infected by Pst DC3000 infection. Water – control, Chitosan – positive control, S223 – extract of lobster shells digested by S223, S224 – extract of lobster shells digested by S224, Shells – extract of undigested lobster shells.

## Slide 11
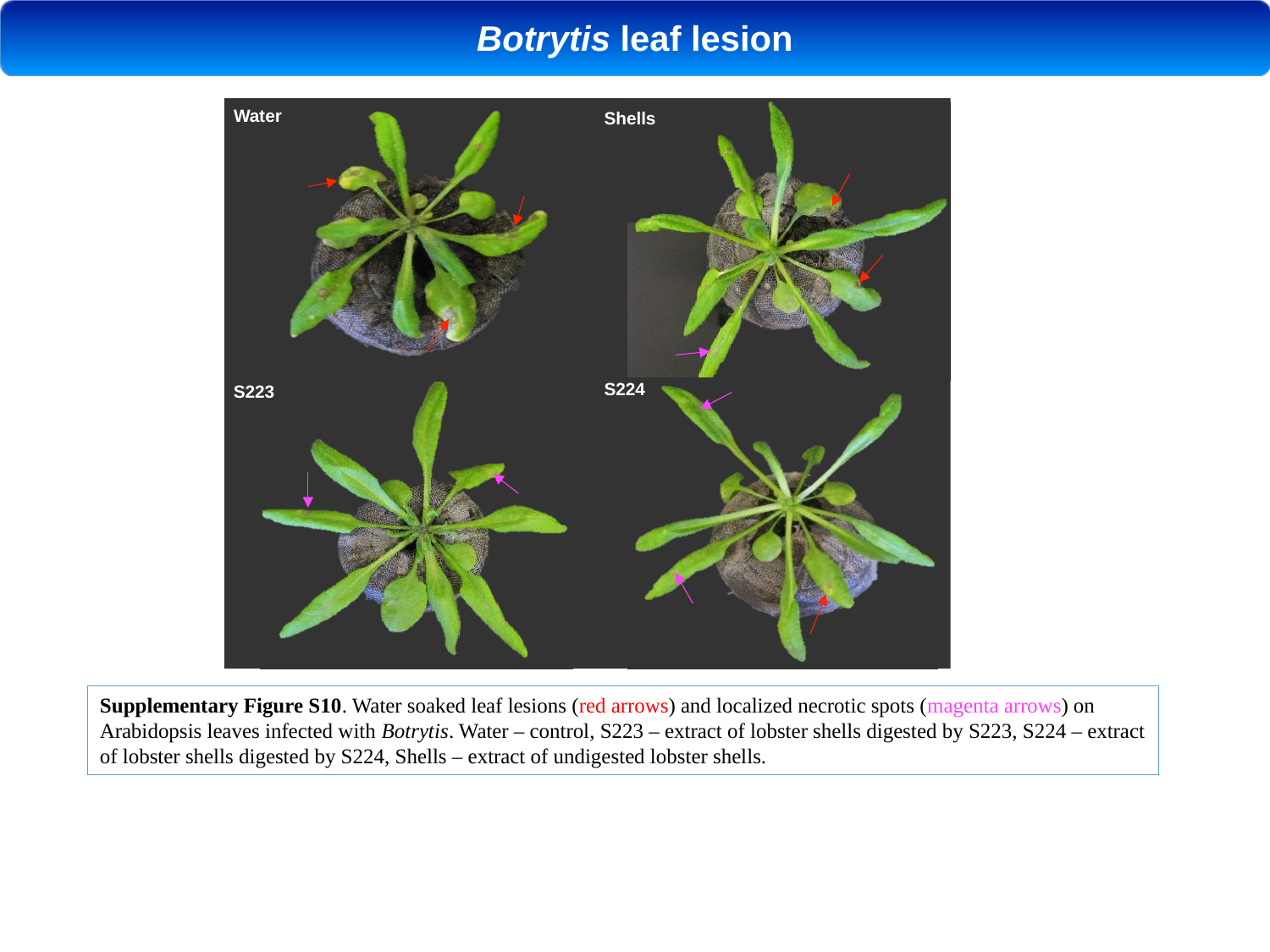

Botrytis leaf lesion
Water
Shells
S224
S223
Supplementary Figure S10. Water soaked leaf lesions (red arrows) and localized necrotic spots (magenta arrows) on Arabidopsis leaves infected with Botrytis. Water – control, S223 – extract of lobster shells digested by S223, S224 – extract of lobster shells digested by S224, Shells – extract of undigested lobster shells.
