# Supplementary material for: Microbial Degradation of Lobster Shells to Extract Chitin Derivatives for Plant Disease Management
Source: Front Microbiol. 2017 May 5;8:781. doi: 10.3389/fmicb.2017.00781 (PMC5418339; doi:10.3389/fmicb.2017.00781)
Supplement: Supplementary file 4 [file Data_Sheet_2.DOCX]

Supplementary Material

Microbial degradation of lobster shells to extract chitin derivatives for plant disease management

**Gayathri Ilangumaran^1^, Glenn Stratton^2^, Sridhar Ravichandran^1^, Pushp S Shukla^1^, Philippe Potin^3^, Samuel Asiedu^2^, Balakrishnan Prithiviraj^1*^**

*** Correspondence:** Balakrishnan Prithiviraj

bprithiviraj@dal.ca

# Supplementary Data

**Data Sheet 1**. Screening microorganisms for their ability to degrade lobster shell based on deproteinization (protease), demineralization (calcium) and chitinolysis (N-Acetylglucosamine).

# Supplementary Figures and Tables

## Supplementary Figures

**Supplementary Figure S1.** Growth of microbes on lobster shell powder agar. (A) Colony diameter of microbes growing on agar plates containing 0.5% (w/v) chitin, cooked or raw lobster shells measured at seven days after incubation. Values represent mean ± SE (n = 3; P < 0.05). (B) Soil isolates spot inoculated on agar plates (i) chitin (ii) cooked (iii) raw lobster shells. *Bs – Bacillus subtilis, Pf – Pseudomonas fluorescens, Lba – Lactobacillus acidophilus, Th – Trichoderma harzianum*

**Supplementary Figure S2.** Calcium content of cooked and fresh lobster shells determined by ash test. The ashen samples of the shells were dissolved in HCl and then fed into a Varian® Atomic absorption spectrometer. Values represent mean ± SE (n = 4). Significant differences are indicated by different letters (P < 0.05).

**Supplementary Figure S3.** Morphological observations of S223 and S224. (A) and (B) Spore bearing filaments (C) and (D) Gram staining (E) and (F) Spore chains of S223 and S224, respectively observed under light microscope (bright field, 40X and1000X magnification).

**Supplementary Figure S4.** Morphological observations of S223 and S224. (A) and (B) are Colonies of S223 and S224 on YEME, oats, starch, glycerol and lobster shell agar plates (left to right) respectively. (C) Spore chains of S223 under 1000X magnification (D) Thioglycollate test (E) Melanin production in peptone-iron and tyrosine agar slants.

**Supplementary Figure S5.** Optimization of temperature and pH for the growth of S223 and S224 observed by measuring colony diameter on day 14 after incubation on agar plates containing raw lobster shells (0.5% w/v). Values represent mean ± SE (n = 3; P < 0.05).

**Supplementary Figure S6.** Detection chitinase in microbial culture filtrates. (A) Dark circular zones indicate chitinase activity on glycol chitin agarose plates, visualized under UV transillumination. Standard plate on the left has serial dilutions of chitinase from *Streptomyces griseus*. (B) Chitinase activity of microbe digested lobster shell extracts, (1) is blank (water), (2) and (3) are culture filtrates of S223 and S224 grown on YEME, respectively, (4) is filtrate from undigested lobster shell powder, (5) and (6) are extracts of S223 and S224 grown on lobster shell powder, respectively.

**Supplementary Figure S7.** Spore germination of *Botrytis* observed 12 h after incubation under light microscope (20X magnification). Water – control, Chitosan – positive control, S223 – extract of lobster shells digested by S223, S223 grown on YEME, S224 – extract of lobster shells digested by S224, S224 grown on YEME, Shells – extract of undigested lobster shells.

**Supplementary Figure S8.** Disease intensity of *Pst* DC3000 infection in Arabidopsis treated with lobster shell extracts measured by visual observation. Water – control, Chitosan – positive control, S223 – extract of lobster shells digested by S223, S224 – extract of lobster shells digested by S224, Shells – extract of undigested lobster shells. Values represent mean ± SE (n = 6; P < 0.05).* significant difference.

**Supplementary Figure S9.** Yellowing symptoms (red arrows) on Arabidopsis leaves infected by *Pst* DC3000 infection. Water – control, Chitosan – positive control, S223 – extract of lobster shells digested by S223, S224 – extract of lobster shells digested by S224, Shells – extract of undigested lobster shells.

**Supplementary Figure S10.** Water soaked leaf lesions (red arrows) and localized necrotic spots (magenta arrows) on Arabidopsis leaves infected with *Botrytis*. Water – control, S223 – extract of lobster shells digested by S223, S224 – extract of lobster shells digested by S224, Shells – extract of undigested lobster shells.

**Supplementary Tables**

**Supplementary Table S1**

**Table S1.** Gene specific primers used to study induced disease resistance in Arabidopsis
